# Elucidation of Novel *cis*-Regulatory Elements and Promoter Structures Involved in Iron Excess Response Mechanisms in Rice Using a Bioinformatics Approach

**DOI:** 10.3389/fpls.2021.660303

**Published:** 2021-06-02

**Authors:** Yusuke Kakei, Hiroshi Masuda, Naoko K. Nishizawa, Hiroyuki Hattori, May Sann Aung

**Affiliations:** ^1^Institute of Vegetable and Floriculture Science, Research Center for Agricultural Information Technology, National Agriculture and Food Research Organization, Ibaraki, Japan; ^2^Faculty of Bioresource Sciences, Department of Biological Production, Akita Prefectural University, Akita, Japan; ^3^Research Institute for Bioresources and Biotechnology, Ishikawa Prefectural University, Ishikawa, Japan

**Keywords:** *cis*-regulatory elements (CREs), motifs, iron toxicity, rice, bioinformatics, promoter sequence, iron, zinc

## Abstract

Iron (Fe) excess is a major constraint on crop production in flooded acidic soils, particularly in rice cultivation. Under Fe excess, plants activate a complex mechanism and network regulating Fe exclusion by roots and isolation in various tissues. In rice, the transcription factors and *cis*-regulatory elements (CREs) that regulate Fe excess response mechanisms remain largely elusive. We previously reported comprehensive microarray analyses of several rice tissues in response to various levels of Fe excess stress. In this study, we further explored novel CREs and promoter structures in rice using bioinformatics approaches with this microarray data. We first performed network analyses to predict Fe excess-related CREs through the categorization of the gene expression patterns of Fe excess-responsive transcriptional regulons, and found four major expression clusters: Fe storage type, Fe chelator type, Fe uptake type, and WRKY and other co-expression type. Next, we explored CREs within these four clusters of gene expression types using a machine-learning method called microarray-associated motif analyzer (MAMA), which we previously established. Through a comprehensive bioinformatics approach, we identified a total of 560 CRE candidates extracted by MAMA analyses and 42 important conserved sequences of CREs directly related to the Fe excess response in various rice tissues. We explored several novel *cis*-elements as candidate Fe excess CREs including GCWGCWGC, CGACACGC, and Myb binding-like motifs. Based on the presence or absence of candidate CREs using MAMA and known PLACE CREs, we found that the Boruta-XGBoost model explained expression patterns with high accuracy of about 83%. Enriched sequences of both novel MAMA CREs and known PLACE CREs led to high accuracy expression patterns. We also found new roles of known CREs in the Fe excess response, including the DCEp2 motif, IDEF1-, Zinc Finger-, WRKY-, Myb-, AP2/ERF-, MADS- box-, bZIP and bHLH- binding sequence-containing motifs among Fe excess-responsive genes. In addition, we built a molecular model and promoter structures regulating Fe excess-responsive genes based on new finding CREs. Together, our findings about Fe excess-related CREs and conserved sequences will provide a comprehensive resource for discovery of genes and transcription factors involved in Fe excess-responsive pathways, clarification of the Fe excess response mechanism in rice, and future application of the promoter sequences to produce genotypes tolerant of Fe excess.

## Introduction

Iron (Fe) is an essential metal for the survival of virtually all living organisms. It is a vital element used by plants for photosynthesis, electron transport, and other redox reactions (Marschner, 1995). It is the fourth most abundant element on Earth, and most soils are rich in Fe. However, its availability to plants is strongly affected by environmental factors including soil type, soil pH, and cultivation conditions (Römheld and Marschner, 1986; Colombo et al., 2014; Masuda et al., 2019). Fe is present in the less-soluble ferric form, Fe(III), and has low availability to plants in aerobic soils, upland cultivation, and high-pH soils. In such environments, all graminaceous plants, including rice, use a chelation strategy (Strategy II), while non-graminaceous plants use a reduction strategy (Strategy I) (Römheld and Marschner, 1986). However, rice is fundamentally grown in flooded anaerobic soils. Under such conditions, Fe(III) is reduced to the highly soluble ferrous (Fe^2+^ ion) form and occurs as ferrous ions in soil, which can be directly taken up by rice roots via ferrous ion transporters such as OsIRT1 and OsIRT2 (Ishimaru et al., 2006). In flooded anaerobic soils, particularly those with low pH, an excess amount of Fe^2+^ ion existed in the soil is absorbed by rice roots, leading to excess Fe accumulation in plant cells. This process causes leaf bronzing and necrosis due to cell death, hindering plant growth and development. Thus, excessive Fe absorption is detrimental to plant growth, and strict regulation of the Fe uptake mechanism at the genetic level is crucial. About 30% of land worldwide and more than 50% of potentially arable land is estimated to have acidic soils (von Uexküll and Mutert, 1995). Rice is a major staple food crop for half of the world’s population. Regions of high rice production such as China, India, and Southeast Asia, west and central Africa are mainly covered with acid soils (NRCS, 2005) in which Fe toxicity is a serious problem (Asch et al., 2005).

The molecular mechanism of the Fe excess stress response in plants is not yet well understood. Recently, comprehensive analyses of Fe excess stress in plants have been conducted using various approaches, including transcriptomics and genome-wide association (Quinet et al., 2012; Finatto et al., 2015; Aung et al., 2018b; Wairich et al., 2020), which have suggested that rice plants employ four defense mechanisms against Fe excess to maintain Fe homeostasis (Aung and Masuda, 2020). In defense 1, rice undergoes rhizospheric oxidization through the formation of Fe plaques on the root surface to avoid massive Fe uptake by roots (Becker and Asch, 2005). The expression of Fe uptake-related transporters such as OsIRT1, OsIRT2, OsYSL2 (NA-Fe(II) transporter), OsYSL15 (DMA-Fe(III) transporter), and OsNRAMP1 (ferrous ion transporter) are strongly down-regulated in roots, preventing Fe uptake by roots at various Fe excess levels by microarray analyses (Quinet et al., 2012; Finatto et al., 2015; Aung et al., 2018b). Moreover, the rice ubiquitin ligases (OsHRZ1 and 2), which are negative regulators of the Fe-deficiency response (Kobayashi et al., 2013), exhibit hypersensitivity to excess Fe in knockdown rice (Aung et al., 2018a). Together, this evidence supports the important role of Fe uptake-related genes and OsHRZ in defense 1 (Fe exclusion from roots) (Aung and Masuda, 2020).

In defense 2, rice may allow excess Fe uptake and then retain it in the roots, preventing Fe translocation from roots to shoots (Tadano, 1975; Becker and Asch, 2005). Next, in defense 3, Fe may be translocated from the roots to shoots and then accumulated in a non-toxic form in vacuoles or as ferritins (Briat et al., 2010). The expression of vacuolar iron transporter (OsVIT2, Zhang et al., 2012) and the Fe storage proteins ferritins (OsFERs, Briat and Lobréaux, 1997; Stein et al., 2009; Briat et al., 2010) increase in roots and shoots of Fe overload plants (Quinet et al., 2012; Finatto et al., 2015; Aung et al., 2018b). Furthermore, the expression of Fe chelator, the nicotianamine (NA) synthase gene (*OsNAS3*) increases markedly in various tissues in response to Fe excess (Aung et al., 2018b) and its knockout plants are sensitive to Fe excess, suggesting an important role of *OsNAS3* in the tolerance mechanism (Aung et al., 2019). It indicates that these genes participate in defense 2 (Fe retention in roots) and defense 3 (Fe compartmentalization in shoots) (Aung and Masuda, 2020). Defense 4 is reactive oxygen species (ROS) detoxification (Aung and Masuda, 2020), wherein rice may resist Fe overload in leaves through enzymatic detoxification or scavenging of ROS with antioxidants (Becker and Asch, 2005; Stein et al., 2009). However, further in-depth characterizations are needed to discover the detailed mechanisms of these adaptation strategies.

Transcription factors are proteins that regulate the expression of genes at the transcriptional level by binding to specific DNA sequences (Mitsis et al., 2020). Therefore, predicting the DNA-binding motifs of the transcription factors is an important component of the functional analyses of transcription factors. *cis*-regulatory elements (CREs) are genomic sequences in promoter regions to which transcription factors bind, and they play a crucial role in precise control of gene expression (Hernandez-Garcia and Finer, 2014). Studies on the transcription factors involved in Fe homeostasis (Fe deficiency and Fe excess responses) have been reported. Kobayashi et al. (2003) identified two Fe-deficiency-responsive CREs, IDE1 (containing a CATGC sequence) and IDE2 (containing a CA[A/C]G[TC][T/C/A][T/C/A] sequence). These CREs bind specifically to the plant-specific transcription factors that regulate Fe homeostasis in rice, IDEF1 (IDE-binding factor 1, ABI3/VP1 family, Kobayashi et al., 2007) and IDEF2 (IDE-binding factor 2, NAC family, Ogo et al., 2008), respectively, which positively regulate the expression of Fe-deficiency-induced genes (Kobayashi et al., 2014). Moreover, another Fe-deficiency-responsive transcription factor, OsIRO2 (bHLH family), binds to a CRE with a CACGTGG sequence and positively regulates Fe-deficiency-responsive genes (Ogo et al., 2007). Furthermore, an Fe-dependent regulatory sequence (IDRS) CCTCCAC sequence has been reported to be responsible for Fe-deficient repression of *ZmFer1* expression in maize and *AtFer1* expression in *Arabidopsis* (Petit et al., 2001). Zhang et al. (2017) reported that OsHRZ1 may be a sensor of the balance between Zn and Fe that interacts with OsPRI1/OsbHLH60. Among the important WRKY superfamily of transcription factors, transcription factors including OsWRKY80, OsWRKY55-like, OsWRKY46, OsWRKY64, and OsWRKY113 have been reported to be Fe excess-induced or related-transcription factors, also in addition to responding to other abiotic stresses such as senescence and drought (Ricachenevsky et al., 2010; Finatto et al., 2015; Viana et al., 2017). Moreover, Viana et al. (2017) suggested that CREs related to abiotic stress, such as light responses and the salicylic acid (SA) pathway, may also be involved in molecular signaling in the Fe excess response. Finatto et al. (2015) analyzed CRE occurrence in putative promoters of Fe excess regulated genes. They reported that CREs related to auxin-responsive element and ABA-responsive elements are present. Maltzahn et al. (2020) reported that the promoters of rice autophagy-related genes (OsATG) are rich in W-box CREs that bind WRKY transcription factors, suggesting the possible involvement of these genes in the early response to Fe toxicity. However, the specific and core CREs in promoter sequences that coordinate the expression of most Fe excess-responsive genes in rice remain unknown. Further promoter analyses of *cis*-acting elements and promoter sequences are essential to understanding Fe excess defense mechanisms in rice.

Today, bioinformatics and machine learning approaches are widely employed to reveal regulatory networks at a genome-wide scale, including high-accuracy prediction of CREs and the genes they regulate. Previous studies have explored CREs related to stress responses in *Arabidopsis* using computational models and machine learning approaches. Zou et al. (2011) identified biotic and abiotic stress-responsive CREs in *Arabidopsis*. Uygun et al. (2019) identified high-salinity-responsive CREs in *Arabidopsis*. Schwarz et al. (2020) identified more than 100 putative CREs associated with the Fe deficiency response in *Arabidopsis* roots. Then, weighted correlation network analysis (WGCNA) can be used to identify modules or clusters of highly correlated genes, summarize those clusters based on the module eigengene or an intramodular hub gene, relate modules to one another and to external sample traits, and calculate cluster membership measures (Langfelder and Horvath, 2008). R version 3.6 (R Core Team, 2017) and WGCNA-R software (version 1.68) can classify gene expression patterns based on transcriptomic data (e.g., Hongwei et al., 2018) through network analyses. Machine learning programs, such as support vector machine (SVM) light (Joachims, 1999), PARTY (version 1.3–5, Hothorn et al., 2008, 2015), and XGBoost version 3 (Chen and Guestrin, 2016), along with the feature selection program Boruta (version 0.3, Kursa and Rudnicki, 2010) have been reported to be efficient applications for the modeling of large datasets. PlantPAN3 (Chow et al., 2019) and PLACE (version 30.0) (Higo et al., 1999) are the largest *cis*-element databases for plants. We also previously developed an innovative bioinformatics method, the microarray-associated motif analyzer (MAMA), for identifying novel *cis*-acting elements based on weighted sequence similarities and gene expression profiles of microarray data (Kakei et al., 2013). In previous report, MAMA precisely simulated more than 87% of gene expression levels after machine learning SVM optimization (Kakei et al., 2013). Particularly, MAMA is an efficient method for precise prediction of CREs in plants subjected to stress. For example, it can identify the CREs responsible for Fe deficiency in rice (*Oryza sativa*) based on the sequences of Fe-deficiency-induced genes (Kakei et al., 2013). In addition, several novel candidate CREs, corresponding to the known *cis*-acting elements ZDRE, ABRE, and DRE, have been identified in zinc-deficient rice and salt-stressed *Arabidopsis* using MAMA (Kitazumi et al., 2018).

Recently, we performed comprehensive morphological, physiological, and transcriptomic (microarray) analyses of hydroponically grown rice subjected to Fe excess stress treatment for 14 days using various levels of Fe excess (× 1, × 10, × 20, × 50, and × 70 Fe) in several tissues (roots, discrimination center [DC, junction notes between root and shoot.], stems, old leaves, and the newest leaves) (Aung et al., 2018b). In this study, to elucidate the regulation of the Fe excess response, we further explored novel Fe excess-responsive CREs and promoter structures in various rice tissues using the microarray database and specific bioinformatics and machine learning approaches, including MAMA. Moreover, we also built a model tree of candidate CRE motifs to simulate the molecular model regulating Fe excess-responsive genes. Our findings may provide to uncover the genes and transcription factors involved in Fe excess-responsive pathways and consequently Fe excess tolerance mechanisms in rice. Moreover, after screening among the investigated promoter sequences, the functional promoters could be further applied to enhance the expression of the target genes for producing Fe excess-tolerant rice.

## Materials and Methods

### Plant Growth and Microarray Data Analyses

**FIGURE 1:**
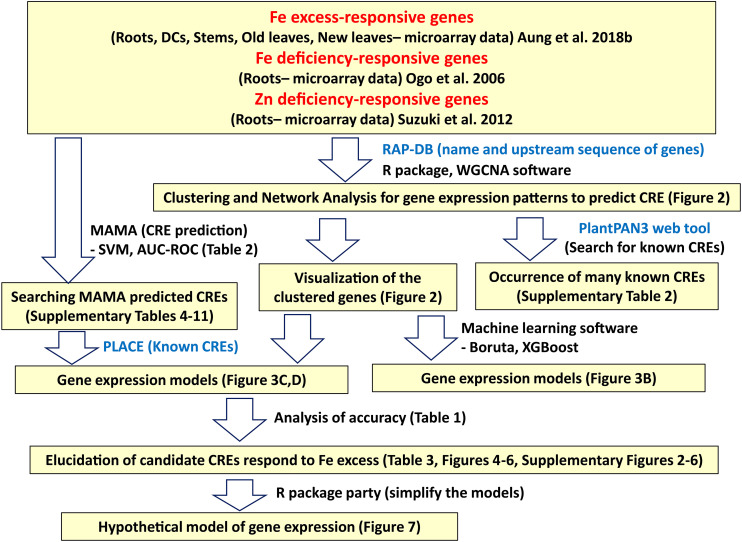
Workflow for the discovery of Fe excess-responsive CREs. Description in the yellow hatched area indicates the data analyzed in each bioinformatics process. The software and methods used to produce bioinformatics data are shown to the right of the arrow.

The microarray data for various rice tissues and Fe excess levels used for bioinformatic analyses were obtained from Aung et al. (2018b). The plant growth conditions were described in Aung et al. (2018b), and can be briefly summarized as follows: 24-day-old rice seedlings (*Oryza sativa* L. cv. Tsukinohikari) were exposed to various ferrous Fe (FeCl_2_) concentrations, including × 10 Fe (0.36 mM), × 20 Fe (0.71 mM), × 50 Fe (1.79 mM), × 70 Fe (2.50 mM) relative to a control solution of × 1 Fe (35.7 μM) for 14 days at pH 4.0 in a modified Kasugai’s hydroponic culture solution. Then, RNA samples were taken from various rice tissues (roots, DC, stems, old leaves [third newest leaves], and newest leaves) under various levels of Fe excess (× 10, × 20, × 50, and × 70 Fe) versus control condition (× 1 Fe), and microarray analyses were conducted using the two-color method. The geometric mean of two biological replicates was used to compare gene expression levels. The rice 44K oligo DNA microarray (Agilent Technologies, Santa Clara, CA, United States) was used to identify Fe-regulated genes, as it contains 43,733 rice DNA probes, covering approximately 24,000 assumed genes and microRNAs based on sequences from the National Institute of Agrobiological Sciences, RefSeq, and Genbank 2007. In this study, the geometric mean gene expression ratios of the Fe-excess groups for each tissue were used for analyses because clustering analyses of the microarray data showed that gene expression patterns under Fe excess ratios of × 10, × 20, × 50, and × 70 were generally consistent (Supplementary Figure 1). Another reason to use mean expression value is to simplify the analyses. Next, the regulation mechanism in Fe excess is closely related to Fe and Zn deficiencies, and Fe excess response genes may show opposite or same behavior patterns associated with Fe-deficient or Zn-deficient condition. Thus, microarray data on Fe-deficient rice roots were obtained from Ogo et al. (2006) and microarray data on Zn-deficient rice roots were obtained from Suzuki et al. (2012). All microarray data used for this study are from microarray analyses by the two-color method which compared stress condition vs normal condition. Fe excess-responsive CREs were elucidated using these microarray data and following the workflow illustrated in Figure 1.

### Network Analyses of Gene Expression Patterns

**FIGURE 2:**
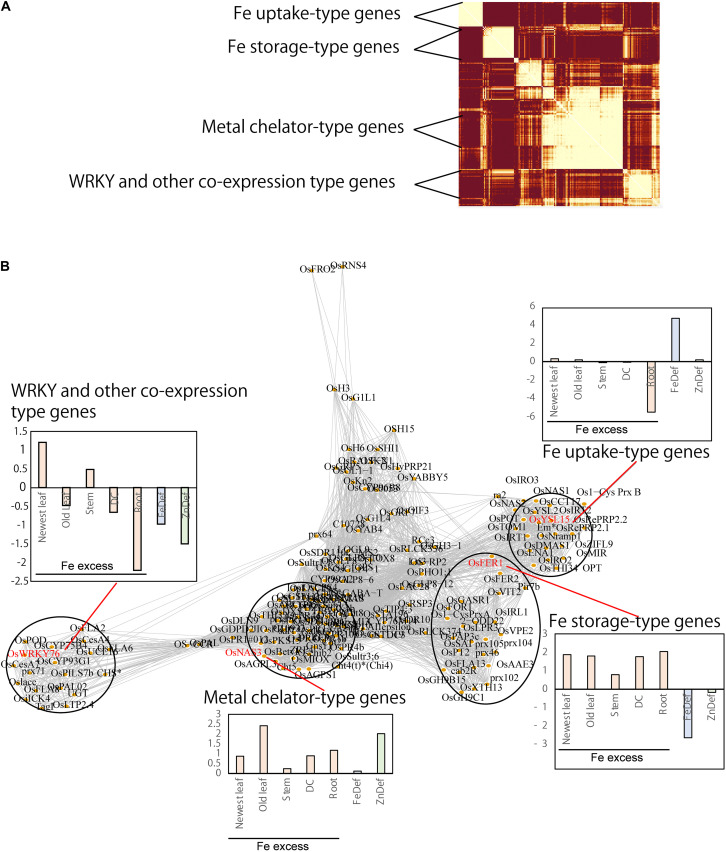
Network analyses of gene expression patterns related to the transcriptional response to Fe excess in each rice tissue type (root, DC, stem, old leaf and newest leaf) analyzed and visualized using the R package WGCNA (weighted correlation network analyses, version 1.68). Differentially expressed genes in four Fe excess treatments (× 10 Fe, × 20 Fe, × 50 Fe, × 70 Fe) were used for this analysis. In addition to the transcriptional response to Fe excess in each tissue, the responses to Fe deficiency and Zn deficiency in rice were also concurrently analyzed. To visualize the clustered genes, only genes with names (under the RAP-DB Gene Symbol system) were used. In this network, similar expression patterns were clustered **(A)**, connected to each other and placed together **(B)**. Expression was largely clustered into four categories. As representative genes for each category, the transcriptional responses (times induced under each stress) of *OsWRKY76* (WRKY and other co-expression), *OsNAS3* (metal chelator), *Ferritin 1* (Fe storage) and *OsYSL15* (Fe uptake) are shown as log2-ratio bar graphs. In each cluster, the gene with a clear role and function in Fe homeostasis are selected as a representative example and shown in red letter.

**FIGURE 3:**
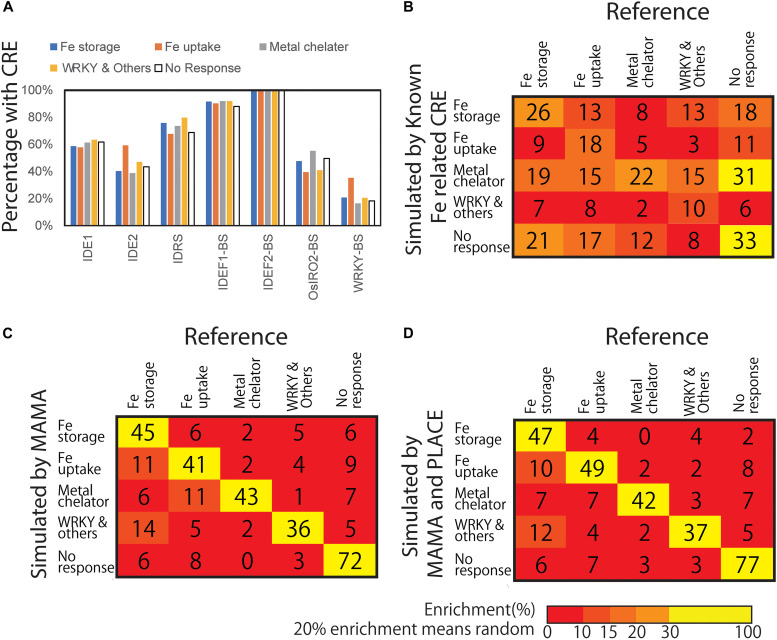
Presence of known CREs and the confusion matrix of gene expression models based on presence and absence of CREs. **(A)** Presence of known Fe-related CREs. Each CRE sequence was searched from 500 bp upstream of the transcription start site (TSS) of each gene included in the Fe response groups. **(B)** Confusion matrix from the gene expression pattern simulation model based on the presence of known Fe-related CREs. **(C)** Confusion matrix of gene expression pattern simulation model based on the presence of MAMA-extracted motifs. **(D)** Confusion matrix of gene expression pattern simulation model based on the presence of MAMA-extracted motifs and CREs recorded in PLACE. Numbers shows the number classified by the model. Color shows Enrichment (%). A total of 20% enrichment equals to random and meaningless model. The higher the diagonal values of the confusion matrix the better, indicating accurate predictions.

Genes that showed more than 2-fold (2^1^) differences in expression between non-treated and treated rice were used for network analyses. However, in the case of Fe-deficient rice roots, genes that differed by more than 2.82-fold (2^1.5^) were considered differentially expressed genes. R software (version 3.6) and the WGCNA package (version 1.68) were used to perform network analyses of gene expression patterns (Figure 2A). For the visualization of clustered genes, representative genes with names in the Rice Annotation Project Database (RAP-DB) gene symbol system are included in Figure 2B. In our other modeling analyses, all named and unnamed genes were included (as shown in Figures 3, 6, 7). The 100 least-affected genes, with differences of less than two-fold, were selected as no-response genes for modeling.

### Analyses of *cis*-Regulatory Element Candidates From Fe Excess-Treated Rice Microarray Data

Transcription factor binding sites (TFBSs), which are shared among the genes of the same expression type, were searched using the PlantPAN3 web tool, an efficient method for investigating critical *cis*- and *trans*-regulatory elements in the promoters of plant genes (Chow et al., 2019). The sequence regions 1,000 bp upstream from of transcription start site (TSS), 100 bp downstream of the TSS, and 500 bp downstream of the transcription end site were set as the search target areas. Then we constructed a tree model (XGBoost version 3; Chen and Guestrin, 2016) to explain the expression patterns in the four Fe excess response clusters and control based on the presence or absence of the Fe-related CREs, IDE1, IDE2, IDRS and binding sequences of IDEF1, IDEF2, OsIRO2, and WRKY (Figures 3A,B). In addition to CREs of Fe excess-responsive genes, we hypothesized that CREs of Fe- or Zn- deficiency-responsive genes might have a new role in Fe excess. Thus, we next explored the new or novel CREs in the Fe excess microarray datasets of Aung et al. (2018b) and the reported or known CREs in the Fe-deficiency or Zn-deficiency microarray datasets of Ogo et al. (2006) and Suzuki et al. (2012), respectively, for their new roles in Fe excess using MAMA version 1.0 Kakei et al. (2013); it is available at https://sourceforge.net/projects/microarray-motif-analyzer/; Source code used for various analyses in this study were shown in Supplementary Data 2–4) by extracting the regulatory sequences in −500 to +150 bp relative to TSS of Fe excess-responsive genes (Figure 3C). Then a gene expression pattern model was constructed from the CREs recorded in PLACE (version 30.0), which is a database of plant CREs (Higo et al., 1999). To obtain highly accurate gene expression simulation, a combined approach of PLACE and MAMA was used (Figure 3D). Genome sequences, TSS, and gene annotations (IRGSP-1.0) were obtained from the RAP-DB website^1^.

### Modeling of Gene Expression Patterns

The R package Boruta (version 0.3; Kursa and Rudnicki, 2010) was used for feature (motif and CRE candidates for modeling) selection. The R package XGBoost (version 1.2.0) was used as the main tree classifier for modeling. The R package rpart (version 4.1–15) was used for the construction of a simple tree model to build a mechanistic model. The R package caret (version 6.0–86) was used to check the accuracy of models by determining the mean of balanced accuracy, area under the receiver operating characteristics curve (AUC-ROC), and confusion matrix (Figures 3B–D). The mean of balanced accuracy was used to optimize the model.

## Results

### Network Analyses of Gene Expression Patterns and Transcriptional Responses to Fe Excess

In general, the expression patterns of genes are tightly linked to CREs existence in their upstream promoter sequences. Thus, we assumed that CREs closely related to Fe excess could be predicted by classifying the expression patterns of Fe response genes and examining the CRE sequences within each expression pattern cluster. Therefore, we performed bioinformatic analyses for identifying Fe excess-responsive CREs based on the workflow shown in Figure 1. We first performed weighted gene co-expression network analyses via WGCNA, and calculated module-trait correlations based on a Fe excess microarray dataset of Aung et al. (2018b). Here, gene expression patterns in each tissue were similar among the × 10, × 20, × 50, and × 70 Fe excess treatments (Supplementary Figure 1). Also, to avoid the complexity with huge data by the analyses of each Fe excess treatment and to make a simple model, in WGCNA analysis, the four levels of Fe excess treatment were merged to obtain a mean expression value for subsequent network analysis. Furthermore, there are several genes that show various gene expression patterns depending on Fe status (Fe excess by Aung et al., 2018b or Fe deficiency by Ogo et al., 2006). Hence, we assumed that the expression patterns of Fe homeostasis-related genes under Fe excess conditions could be better visualized by analyzing the gene expression patterns under Fe deficiency reported by Ogo et al. (2006). Additionally, the behavior of Fe and Zn nutrition in rice are closely related, and likely involve similar regulation processes of gene expression (Suzuki et al., 2012; Masuda et al., 2020). OsHRZs and IDEF1 regulate downstream genes by sensing the balance between Zn and Fe availability (Kobayashi et al., 2013). Therefore, we compared transcriptional response data from microarray analyses of Fe-deficient and Zn-deficient rice roots, in addition to tissue-specific data from rice plants under Fe excess conditions (Figure 2).

Interestingly, network analyses of Fe excess-regulated genes showed that they can be largely categorized into four expression clusters, namely the Fe storage, metal chelator, Fe uptake, and WRKY and other co-expression types (Figure 2A). The identification codes (gene locus ID) and names of the genes in each type are listed in Supplementary Table 1. Genes categorized as Fe storage type were up-regulated under Fe excess conditions in all tissues and down-regulated in the root under Fe-deficiency treatment (Figure 2B). Fe storage type genes include the Fe storage-related *ferritin* genes *OsFer1*, *OsFer2*, and the vacuolar processing enzyme gene *OsVPE2*. Genes categorized as metal chelator type were up-regulated under Fe excess and Zn-deficiency treatments. This group includes the *OsNAS3*, *OsPR1*, and *OsPR4* genes. OsNAS3 plays a role in the synthesis of nicotianamine, a chelator of Fe and Zn. Genes categorized as Fe uptake type include *TOM* (DMA efflux transporter; Nozoye et al., 2011), *OsIRT1*, *OsYSL2*, *OsYLS15*, and *OsNRAMP1*. Genes categorized as WRKY and other co-expression type include stress response genes such as OsWRKY76.

### Prediction of Fe Excess-Related CREs Through Machine Learning Approaches

Genes with similar expression patterns may be regulated by the same transcription factor and molecular mechanism, and therefore may share the same CRE set. To investigate whether CREs are shared among genes in the same expression type, TFBSs that are shared among genes with the same expression type were explored using an efficient machine learning method, the PlantPAN3 web tool (Chow et al., 2019). The sequences 1,000 bp upstream of the TSS, where most CREs are occurred, 100 bp downstream of the TSS, and 500 bp downstream of the transcription end site were set as the target areas. We found that 93% of Fe storage type genes shared binding sequences of the TBP, AP2, AT-Hook, NF-YB, TCP, homeodomain, B3, bZIP, or alpha-amylase transcription factors (Supplementary Table 2). On the other hand, more than 90% of the least-regulated sequences (which did not regulate downstream genes) shared the AT-Hook, NF-YB, TCP, homeodomain, B3, bZIP, SBP, C2H2, or bHLH transcription factor binding sequences (Supplementary Table 3).

To explore the presence of CREs in Fe excess-responsive genes more specifically, reported Fe-associated CREs, i.e., the binding sequences of the transcription factors IDEF1, IDEF2, IRO2, and WRKY and the promoters IDS1, IDS2, and IDRS, were used as search queries against sequences 500 bp upstream of the TSS for genes in each expression cluster type based on Fe excess conditions (Figure 3) and for genes whose expression is not regulated by Fe excess treatment. This query 500 upstream of TSS is reported to have more transcription factor-binding motifs in plants (Weirauch et al., 2014). The percentage of genes with each CRE in the 500 bp upstream region are shown in Figure 3A. The results showed that Fe uptake type genes contained more IDE2 and WRKY binding sequences in their upstream regions than genes showing no response. IDRS was enriched at the upstream of Fe storage-, metal chelator- and WRKY-type genes. However, enrichment of other CREs in the promoter sequence regions was not significantly elevated among Fe excess-responsive genes by Fisher’s exact test. Overall, the presence of these CREs alone was not specific to Fe excess-related genes.

Next, we constructed a tree model (XGBoost version 3; Chen and Guestrin, 2016) to explain expression types (four Fe excess response types and the no response type) based on the presence or absence of Fe-related CREs. Ksouri et al. (2021) reported that motif extraction was successful using −500 to +200 regions. MAMA has a function to extract motifs from −500 to +150 region (Kakei et al., 2013). Therefore, the search target of motifs was set as −500 to +150 relative to TSS in the following analyses. The tree model classified the data using a combination of criteria, namely, the combination of CREs present, to classify the expression patterns. Figure 3B shows the confusion matrix of gene expression pattern simulation model resulting from classification simulation with a constructed model based on the presence of combinations of reported or known CREs (Fe-related CREs including IDE1, IDE2, IDRS, OsIRO2- and WRKY- binding site motifs, etc.). For example, among the total 78 genes considered Fe storage type genes in this simulation, only 26 were actually categorized as Fe storage type genes based on microarray data (Figure 3B). The remaining 13, 8, 13, and 18 genes were categorized as Fe uptake type, metal chelator type, WRKY and other co-expression type, and no response type, respectively. If the simulation is completely random and shows no difference, all genes are categorized as 20% each and none will be enriched. This tree model based on reported Fe-related CREs explained only 56.9% of gene expression patterns as balanced accuracy (average of specificity and sensitivity) (Table 1). This parameter is useful when evaluating a classification model of imbalanced data. The whole result of statistical data including specificity and sensitivity is shown in Supplementary Data 1. The confusion matrix showed that gene expression patterns simulated with this tree model were generally inconsistent with the referenced data (Figure 3B). This finding indicates that the use of only reported Fe-related CREs was insufficient to explain the overall expression patterns of Fe excess-responsive genes. Therefore, we hypothesized that other CREs exist, which are specific to Fe excess-responsive genes rather than to known Fe-homeostasis genes.

**TABLE 1:** Accuracy of the gene expression pattern simulation model.

Motifs used for model	Accuracy
Iron-related CREs	56.9%
Known CREs in PLACE	66.8%
MAMA-predicted candidates	80.9%
Place and MAMA	83.0%

*Accuracies of Boruta-XGBoost models are listed. In this model, genes were classified based on the presence/absence of motif sequences (known CREs and CRE candidates). Iron related CREs are the binding sequences of the transcription factors IDEF1, IDEF2, IRO2, and WRKY and the promoters IDS1, IDS2, and IDRS. 469 motifs are recorded in PLACE database as known. MAMA extracted 560 CRE candidates including duplicated.*

Thus, to explore novel Fe excess CREs and obtain a better mechanistic understanding of the Fe excess response and the sequence of upstream genes involved, the CRE prediction tool MAMA (Kakei et al., 2013) was applied to microarray data from Fe excess-treated newest leaves, old leaves, stem, DC, and root samples. Based on the resulting CRE predictions, we found that MAMA-predicted CREs were specifically enriched among Fe excess-regulated genes, in contrast to the results obtained from PlantPAN3 (Supplementary Figures 2–6). At the same time, MAMA constructed a gene expression model, which showed whether genes are regulated or not by the predicted CREs. This MAMA simulation accurately classified genes as Fe excess-regulated genes with CRE candidates or as non-responsive genes. The accuracy of the MAMA simulation (SVM classification model of the Fe excess response in each tissue based on the presence or absence of predicted CREs) is described in Table 2. The accuracy of the Fe excess response for all tissues was more than 86% (Table 2). The models showed that more than 86% of randomly selected and under sampled test data was accurately simulated in 5 times cross validation. Table 2 also provides accuracy and AUC-ROC values for the Fe excess response simulation model. AUC-ROC generally ranged between 1.0 (highest, indicating perfect specificity) and 0.5 (not specific, random). The average AUC-ROC value of the simulation models was 0.77. We considered these accuracy and specificity (AUC-ROC value) estimates sufficiently high to support use of the MAMA program to build a hypothetical model.

**TABLE 2:** Accuracy of the Fe excess response simulation model in each tissue.

Tissues	Accuracy	AUC-ROC
Newest leaf	86.0%	0.75
Old leaf	88.3%	0.80
Stem	86.3%	0.73
DC	93.1%	0.79
Root	88.5%	0.80

*MAMA builds SVM model to classify genes to responsive or not. The model is based on the presence/absence of extracted CRE candidates. In this study, CRE candidates were extracted from Fe-excess treated microarray data in each tissue. This table describes the accuracy of MAMA classified genes in each referenced real data. In addition to check the specificity of the model AUC-ROC value (1.0 means specific and 0.5 means not specific).*

Using microarray data of Fe excess-treated rice tissues, MAMA suggested about 50 expression-related CRE candidates from 500 bp upstream and about 30 CRE candidates from −50 to +150 of TSS in each tissue (Supplementary Tables 4–8). In addition to these CRE candidates identified from newest leaves, old leaves, DC, stems, and roots of Fe excess-treated rice (Aung et al., 2018b), CRE candidates were extracted from microarray data of Fe-deficient (Ogo et al., 2006) and zinc-deficient (Suzuki et al., 2012) rice roots (Supplementary Tables 9, 10). In this manner, a total of 560 CRE candidates were extracted using MAMA (Supplementary Tables 4–10). Then, the list of motifs was further narrowed using the Boruta all-relevant feature selection method (Kursa and Rudnicki, 2010), which can identify important motifs for machine learning models. This selected 30 CRE candidate MAMA motifs in the 500 bp sequence upstream of the TSS (Supplementary Table 11). Top 10 CRE candidates were IDEF1 binding (CATGCATG), Downstream core promoter element in plant 2 (DCEp2: ATCGATCG), Novel motif extracted from Zn deficiency-responsive genes (ATAATGGC), Novel GCWGCWGC motif, Novel CGACACGC motif, Novel Myb binding-like motif CACCAACC, Zinc finger binding motif GCGCGCCA and bZIP/bHLH binding motif CTACGTGC. The importance (weight in tree model) of these CREs are described in Table 3 and Supplementary Table 11. Then, the XGBoost tree model was constructed based on the MAMA-extracted CRE candidates. The confusion matrix of the gene expression pattern simulation model showed good consistency between the number of genes categorized through simulation and those categorized from real microarray data (Figure 3C). For example, 45 genes (70.3%) were categorized as Fe storage genes from referenced microarray data, among a total of 64 genes considered Fe storage type genes for simulation (Figure 3C). This ratio is clearly higher than that based on the presence of known Fe-related CREs shown in Figure 3B. The tree model based on MAMA motifs correctly explained a total of 80.9% of gene expression patterns (Figure 3C and Table 1).

**TABLE 3:** Important motifs in the gene expression pattern model.

Sequence	Importance (weight in tree model)	Motif Name and Annotation
CATGCATG	100.00	IDEF1 binding
ATCGATCG	56.83	Novel: Downstream core element in plant 2 (DCEp2)
ATAATGGC	54.71	Motif extracted from Zn regulated genes at downstream of TSS
GCAGCAGC	54.05	Novel: GCWGCWGC
CGACACGC	49.93	Novel: CGACACGC
EECCRCAH1	48.68	Myb- binding
CACCAACC	48.68	Novel: Myb-binding like
CRTDREHVCBF2	48.22	AP2/ERF binding
GCGCGCCA	46.23	GCGC box
CTACGTGC	44.20	bZIP/bHLH

*Top 10 the most important motifs in Boruta-XGBoost model based on MAMA and PLACE model. Importance was calculated as xgboost feature importance to be between 0 (least important) and 100 (most important).*

CREs which previously identified for stress response such as SA-related CREs and WRKY-related CREs, also play roles in the Fe excess response (Viana et al., 2017). Therefore, we constructed a gene expression pattern model based on both MAMA and CREs recorded in PLACE (version 30.0), a database of plant CREs (Higo et al., 1999). Figure 3D shows the confusion matrix of the gene expression pattern simulation model by the Boruta-XGBoost tree model using MAMA CREs and PLACE CREs. It showed better consistency between the number of genes categorized through simulation and those categorized from actual microarray data (Figure 3D). We compared the accuracy of Boruta-XGBoost models based on various CREs and found that Fe-related (reported) CREs had 56.9% accuracy, PLACE CREs had 66.8% and MAMA motifs had 80.9% (Table 1). The maximum accuracy was obtained using both PLACE and MAMA, at 83.0% which is the highest (Table 1).

### Important CRE Candidates Located Upstream of Fe Excess-Responsive Genes

**FIGURE 4:**
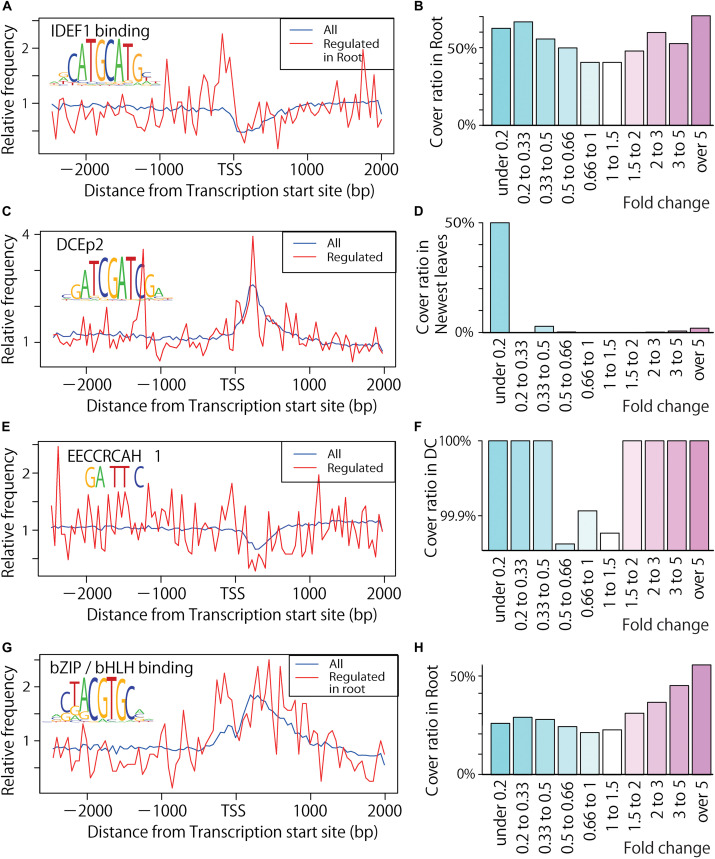
Distribution of important CRE candidates in upstream sequences of Fe excess-responsive genes. **(A)** Distribution of the CATGCATG (IDEF1 binding) motif in all genes and in Fe excess-regulated genes of roots. Blue line shows all genes and red line shows the Fe excess-regulated genes. **(B)** Coverage ratio (percentage of genes including motif among the up- or down-regulated genes by Fe excess as fold changes described under graph) of the CATGCATG motif in the –500 bp to +150 bp area relative to the transcription start site (TSS) in Fe excess-treated rice roots. **(C)** Distribution of the ATCGATCG (DCEp2) motif in Fe excess-treated newest leaves. **(D)** Coverage ratio of the ATCGATCG motif in Fe excess-treated rice newest leaves. **(E)** Distribution of the GANTTNC motif (EECCRCAH1) in Fe excess-treated DC. **(F)** Coverage ratio of the GANTTNC motif (EECCRCAH1) in Fe excess-treated rice DC. **(G)** Distribution of CTACGTGC (bZIP/bHLH binding) motif in Fe excess-treated root. **(H)** Coverage ratio of the CTACGTGC (bZIP/bHLH binding) motif in Fe excess-treated rice roots. Graphs **(A,C,E,G)** show relative frequency compared to the percentage frequency in each 50 bp window within –3,000 to +2,000 bp of the TSS. Graphs **(B,D,F,H)** show coverage ratio of the motifs in the -500 bp to +150 bp area relative to the TSS in Fe excess-treated tissues. Numbers below the graphs **(B,D,F,H)** indicate gene expression ratios in Fe excess-treated rice relative to non-treated rice. DC means discrimination center or junction nodes between root and shoot.

**FIGURE 5:**
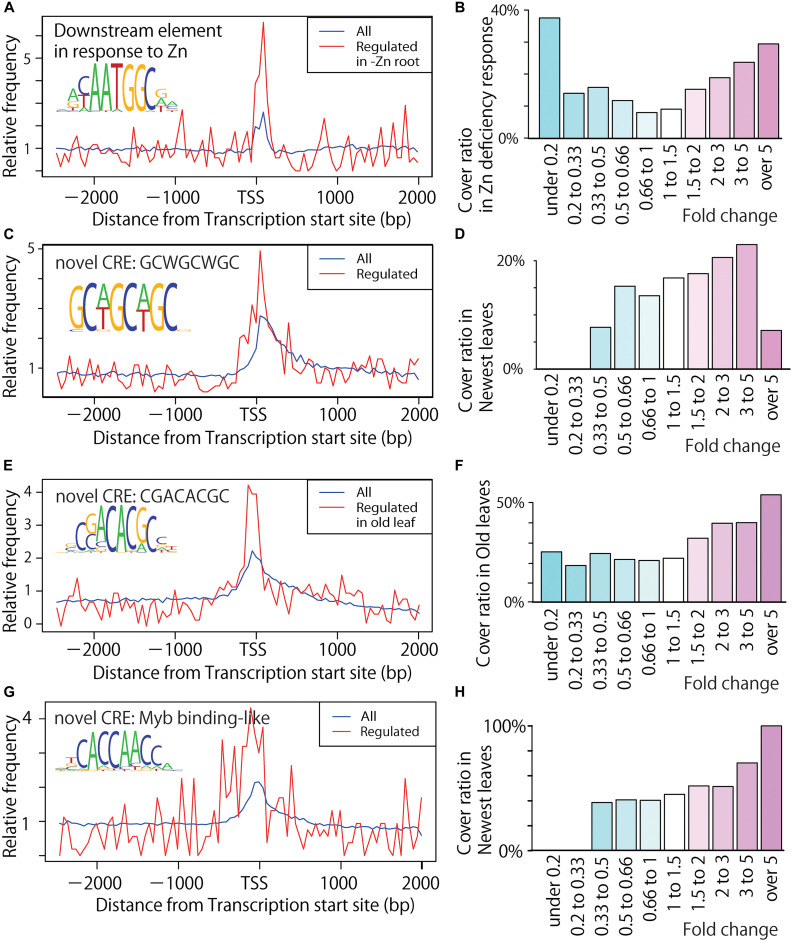
Distribution of novel CRE candidates upstream of Fe excess-responsive genes. **(A)** Distribution of the ACAATGGC motif (novel) in all genes and Zn deficiency-regulated genes in root tissue. Blue line shows all genes and red line shows the Fe excess-regulated genes. **(B)** Coverage ratio (percentage of genes including motif among the up- or down-regulated genes by Zn deficiency as fold changes described under graph) of the ACAATGGC motif (novel) in the –500 bp to +150 bp area relative to the transcription start site (TSS) of Zn deficiency-treated root tissue. **(C)** Distribution of the GCWGCWGC motif (novel) in Fe excess-treated newest leaves. **(D)** Coverage ratio of the GCWGCWGC motif (novel) in newest leaves. **(E)** Distribution of the CGACACGC motif (novel) in Fe excess-treated old leaf tissue. **(F)** Coverage ratio of the CGACACGC motif (novel) in Fe excess-treated old leaf tissue. **(G)** Distribution of the Myb binding-like novel motif (CACCAACC) in Fe excess-treated newest leaves. **(H)** Coverage ratio of the Myb binding -like novel motif (CACCAACC) in Fe excess-treated newest leaf tissue. Graphs **(A,C,E,G)** show relative frequency compared to the average frequency in each 50 bp window within –3,000 to +2,000 bp of the TSS. Graphs **(B,D,F,H)** show coverage ratio of the motifs in the -500 bp to +150 bp area relative to the TSS in Fe excess-treated tissues. Numbers below the graphs **(B,D,F,H)** indicate gene expression ratios in Fe excess- or Zn deficiency-treated rice compared to non-treated rice.

We identified 560 CRE candidates, including known or novel CREs, extracted using MAMA analyses (Supplementary Tables 4–10). We obtained known CRE sequences, which are related to Fe excess response, from MAMA analyses and the plant CRE database PLACE (Figure 4 and Table 3). Then, among these CRE candidates, we selected 42 important motifs through Boruta-XGBoost gene expression simulation, as described above. From this simulation, we identified the most important CREs identified by MAMA, which were annotated as IDEF1 binding (CATGCATG), DCEp2 (ATCGATCG), EECCRCAH1 (GANTTNC), and bZIP/bHLH binding (CTACGTGC) (Table 3). EECCRCAH1 was important to classify no response genes. From our results, the distributions of these known CREs important CRE candidates in upstream regions of Fe excess-responsive genes are illustrated in Figure 4. Novel CREs including ACAATGGC (putative Zn-responsive CRE), GCWGCWGC, CGACACGC, and CACCAACC (Myb binding-like) were important for improving model accuracy (Figure 5). The other important CREs are illustrated in Supplementary Figures 2–6. The distributions of these important motifs in all genes and Fe excess-regulated genes in specific tissues were determined based on relative frequency in the region of −3,000 bp to +2,000 bp relative to the TSS (Figures 4A,C,E,G, 5A,C,E,G). Moreover, the appearance of these important motifs in genes induced or suppressed to varying degrees (considering induced as a change from 1 to over 5, and suppressed from 1 to under 0.2) are illustrated using the coverage ratio (percentage of sequences including motif) of these important motifs in the region −500 bp to +150 bp relative to the TSS in specific tissues (Figures 4B,D,F,H, 5B,D,F,H).

For example, the distribution of the IDEF1-binding CRE (CATGCATG) was determined in roots upstream of Fe excess-regulated genes and in all genes (Figure 4A). These data indicate that IDEF1-binding CREs are two times more frequent between −200 and 0 bp of the TSS in Fe excess-induced genes compared to all other genes. Such motifs appeared frequently, occurring in approximately 50% of genes induced two-fold and 70% of the genes induced by over five-fold (Figure 4B). This CRE occurred in only 40% of genes unregulated by Fe excess (between 0.66 to 1.5 times) but in more than 50% of genes regulated by Fe excess (with changes greater than 2-fold or less than 0.5-fold). The DCEp2 CRE (ATCGATCG) was found in the proximal downstream region of the TSS of Fe excess-regulated genes in the newest leaf (Figure 4C), and it presented at about 50% of Fe excess-downregulated genes less than 0.2 fold, compared to only a few percentage of genes unregulated by Fe excess (Figure 4D). Moreover, EECCRCAH1 (TTATTT) was relatively common (100%) in the upstream sequences of Fe excess-regulated genes (Figures 4E,F) while no response genes occasionally do not have the motif. A CRE bZIP/bHLH binding (CTACGTGC) sequence is related to the Fe excess response in roots (Figures 4G,H).

**FIGURE 6:**
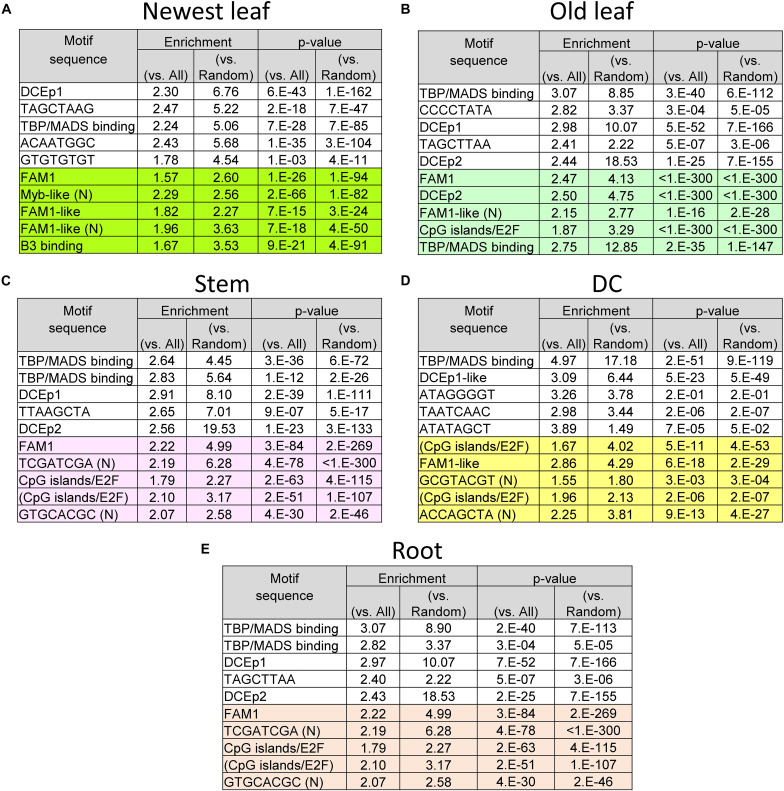
List of extracted CRE candidates from each tissue: **(A)** Newest leaf, **(B)** Old leaf, **(C)** Stem, **(D)** DC and **(E)** Root. DC means discrimination center or junction notes between root and shoot. White background cells show CRE candidates from –50 to +150 window, colored background shows CRE candidates from 500 bp upstream of TSS. (N) indicates CRE candidates that were not reported before. CpG islands/E2F (CGCGCGCA) motif has variants CGCGCGCG, CGCGCGTA, and CGCGCGTG. They were annotated as “(CpG islands/E2F).” Enrichment of motifs were calculated as (number in regulon)/(total length of 500 bp upstream or –500 bp to +150 bp sequence of regulon) divided by (number in all genes)/(total length of upstream 500 bp sequence of all genes). *P*-value was calculated as binominal test using number of motifs found in regulon, number of regulon genes, chance of motif found in all genes. The enrichment and *p*-value were also calculated using the same size of random sequence instead of sequence of all genes.

The MAMA-extracted novel CRE candidates ACAATGGC (putative -Zn responsive CRE), GCWGCWGC, CGACACGC and Myb binding-like (CACCAACC) are distributed in the proximal region of the TSS of Zn deficient roots, Fe excess-regulated genes in the newest leaves and old leaves (Figures 5A,C,E,G), and the motif appears in about 10% more of genes induced by over three-fold (Figures 5B,D,F,H). Other important MAMA-identified motifs located upstream of Fe excess-responsive genes in various rice tissues were investigated as known or novel CRE candidates; these motifs were CACCAACC (novel: Myb binding-like), FAM1 (AGCTAGCT), and ACACACTC (novel) in the newest leaves (Figure 6A, Supplementary Figure 2, and Supplementary Table 4); FAM1 (AGCTAGCT), GATCGATC (novel), and GCATGCAC (novel) in old leaves (Figure 6B, Supplementary Figure 3, and Supplementary Table 5); FAM1 (AGCTAGCT), TCGATCGA (novel), TGCACGC (novel), and CpG islands/E2F (CGCGCGTG) in stems (Figure 6C, Supplementary Figure 4, and Supplementary Table 6); FAM1 (AGCTAGCT), AGCTAAGCT (novel), GATCGATC (novel), and CpG islands/E2F (CGCGCGCG) in DCs (Figure 6D, Supplementary Figure 5, and Supplementary Table 7); and FAM1 (AGCTAGCT), ATCGATCG (novel), and CpG islands/E2F (GCGCGCCA) in roots (Figure 6E, Supplementary Figure 6, and Supplementary Table 8).

### Hypothetical Modeling of the Fe Excess-Responsive Transcriptional Regulation

**FIGURE 7:**
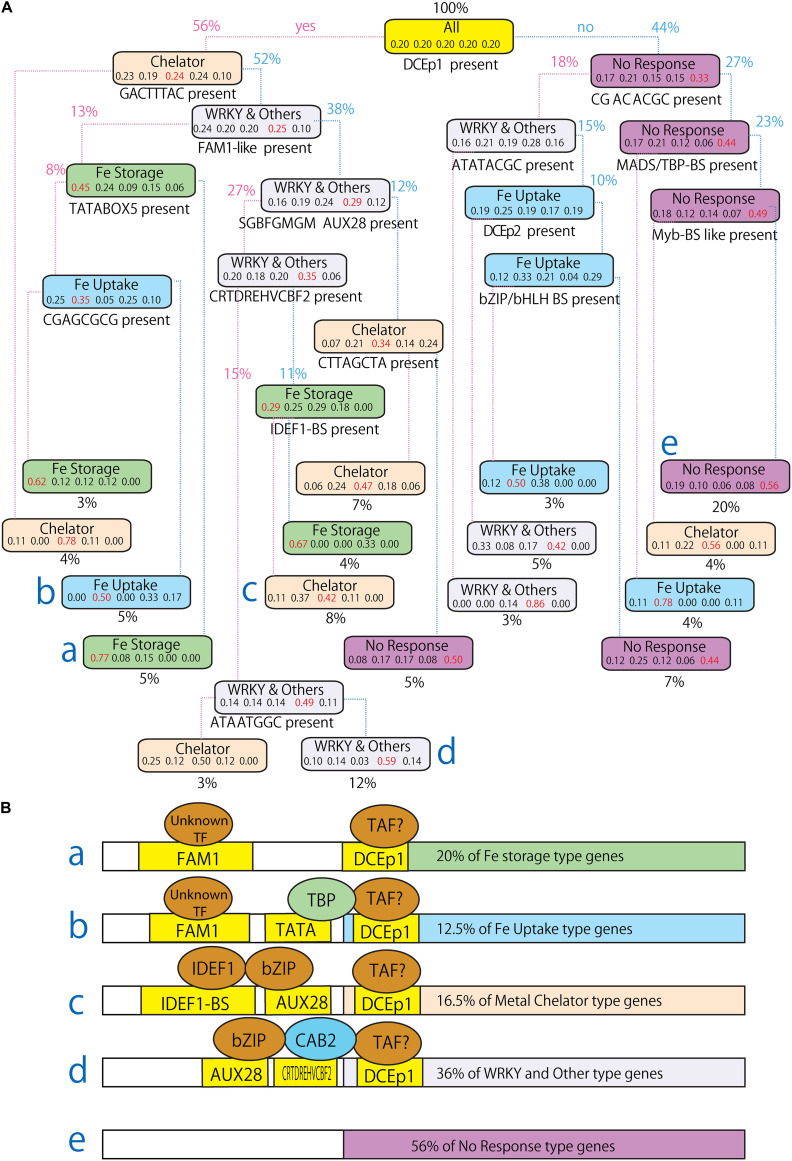
Hypothetical model revealing the underlying Fe excess-responsive transcriptional regulation. **(A)** Simple tree model and YES-NO-Flowchart to explain Fe excess-responsive gene expression patterns. CRE candidate motifs were searched in the regions –500 bp to +150 bp relative to TSS of genes to classify gene expression patterns and each gene is divided by YES or NO flowchart by whether specific CRE candidate motifs described under the box is present or not. The percentage side of the flowchart line indicates the percentage of genes which present (YES) or absent (NO) the CRE shown above. The five numbers shown in each box indicate the percentage of genes belongs in the (left to right) Fe storage type, Fe uptake type, chelator type, WRKY and other response type or no response type patterns. The name of a representative gene expression type is provided in each box. **(B)** Representative molecular model for the regulation of Fe excess-responsive genes based on the above simple tree model. Each molecular model **(a–e)** represents the simulation output (marked as **a–e**) in the above tree model, respectively. In this hypothesis, the transcription factor IDEF1 binds to CATGCATG, bZIP binds to SGBFGMGMAUX28, TBP binds to CTATAAAT, and CAB2 binds to CRTDREHVCBF2. The transcription factors (TFs) that bind to CTACACCT, FAM1, and DCEp1 remain unknown and they are shown as “Unknown TF.” TAF, TBP associated factor. The percentage was calculated as follows: ((Percentage of specific type genes in specific branch) × (percentage of all types in specific branch))/(percentage of specific type (20%)/percentage of all types (100%)). For example, 12.5% of Fe uptake type genes described in B model was calculated as follows: (0.05 * 0.50)/(20/100) = 0.125.

To identify the model of transcriptional regulation and promoter structures for Fe excess response, combinations of CREs involved in transcriptional regulation under Fe excess were analyzed. The tree model generated using Boruta-XGBoost is complicated and does not indicate a straightforward hypothetical model. A tree model built using the R package party (version 1.3–5) is much simpler and easier to understand than that from the XGBoost model, although it is less accurate. Thus, based on the important motifs which identified using the Boruta selection method, in this study, we generated a tree model (YES-NO-Flowchart) using the party package (Figure 7A). This model shows the percentage of each gene expression pattern associated with the presence or absence of CRE elements. Five numbers are shown in each box, indicating the percentage of the genes with specific motif categories: Fe storage type, Fe uptake type, chelator type, WRKY and other co-expression type, and no response type. Starting from all undersampled genes that contain 20% of each type, for example, the DCEp1 motif (AGCTAGCT) is present in 56% of genes and absent from 44% of genes. The 56% of genes containing this motif is highly enriched (24%) in chelator synthesis type genes, while the remaining 44% of genes are most enriched (33%) in no-response genes. The DCEp1 motif (AGCTAGCT) and GACTTTAC are both present in 4% of genes, while 52% of genes contain DCEp1 motifs (AGCTAGCT) and no GACTTTAC motif. The average accuracy of this tree in cross-validation was 68.4% (confusion matrix not shown).

From this tree model, we generated a hypothesis of the molecular model of promoter structures regulating the five types of Fe excess-related gene expression patterns (Figure 7B). Fe storage type genes tended to simultaneously contain the DCEp1 motif (AGCTAGCT), and FAM1 motifs. Fe uptake type genes tended to have FAM1-like motif (ACGTACGC), TATABOX5 and DCEp1 motif (AGCTAGCT). Metal chelator synthesis type genes tended to have DCEp1 motif (AGCTAGCT) and IDEF1-binding motif (CATGCATG). WRKY and other co-expression type genes generally contained the DCEp1 motif (AGCTAGCT), bZIP-binding SGCFGMGMAUX28 motif, and CAB2 binding CRTDREHVCBF2 motif. The genes that did not respond to Fe excess did not include any motif listed above.

## Discussion

### Fe Excess Co-Expression Clusters Based on Network Analyses

The workflow used for elucidation of Fe excess-responsive CREs is presented in Figure 1. Firstly, the four levels of Fe excess treatment (× 10 Fe/ × 1 Fe, × 20 Fe/ × 1 Fe, × 50 Fe/ × 1 Fe, × 70 Fe/ × 1 Fe) were merged for WGCNA analysis to obtain a mean expression value for subsequent network analysis in Figure 2. In this study, four Fe excess levels for WGCNA analysis were not differentiated to simplify the MAMA analyses. Then, we performed network analyses to predict Fe excess-related CREs through categorization of the expression patterns of Fe-responsive gene types and examination of the CRE sequences in each cluster using the microarray dataset of Fe excess-treated rice from Aung et al. (2018b). In addition, we used microarray data of Fe-deficient and Zn-deficient roots by the following reasons. To perform network analysis in Figure 2, we hypothesized that there are several genes that show various gene expression patterns depending on Fe status based on the microarray results of Fe deficiency by Ogo et al. (2006) and Fe excess by Aung et al. (2018b). In addition, it has been found that Fe excess condition causes Zn deficiency in plants, and there was an increased expression of the genes involved in Zn absorption (Aung et al., 2018b). Moreover, the gene regulation process of Fe and Zn nutrition are strongly related (Suzuki et al., 2012; Masuda et al., 2020). Thus, we thought that by taking into consideration the Fe-deficient and Zn-deficient microarray data in the analyses of Fe excess data using MAMA, it can be revealed the links between control of the Fe excess, Fe deficiency and Zn deficiency responses, along with their upstream transcription factors.

From this network analysis, interestingly, we found four major gene expression type clusters, namely, Fe storage type, metal chelator type, Fe uptake type, and WRKY and other co-expression type (Figure 2 and Supplementary Table 1). As shown in Figure 2, Fe storage type genes were up-regulated under Fe excess in all tissues and down-regulated under Fe deficiency in the root. Fe storage type genes include *OsFER1* and *OsFER2*, which are important for Fe storage and detoxification in rice (Stein et al., 2009). Metal chelator and synthesis type genes were up-regulated with Fe excess and Zn-deficiency treatments. Fe excess leads to Zn deficiency and a decrease in Zn concentration was proportionately related to an increase in Fe concentration in roots (Figure 2, Aung et al., 2018b). This expression type of metal chelator includes *OsNAS3* genes involved in synthesis of nicotianamine, a metal chelator of Fe and Zn in higher plants. *OsNAS3* expression was elevated in roots and shoots of rice under Fe excess (Aung et al., 2018b) or Zn-deficient conditions (Suzuki et al., 2008). *OsNAS3* expression is important for detoxification of Fe excess and metal transport (Aung et al., 2019). Next, genes categorized as Fe uptake type were suppressed in Fe excess-treated rice, particularly in roots, and up-regulated in Fe-deficiency-treated rice roots (Figure 2). Fe uptake type genes include the Fe uptake-related genes *TOM*, *OsIRT1*, *OsYSL2*, *OsYSL15*, and *OsNRAMP1*, which are down-regulated in roots under Fe excess (Aung et al., 2018b) and up-regulated in Fe-deficient roots (Ogo et al., 2006). WRKY and other co-expression type genes include stress response genes such as *OsWRKY76*, which were up-regulated in stems under Fe excess (unpublished data and microarray data of Aung et al., 2018b) and down-regulated in Fe-deficient roots (Ogo et al., 2006). Some WRKY TFs are involved in the Fe toxicity response in rice (Ricachenevsky et al., 2010; Finatto et al., 2015; Viana et al., 2017). WRKY transcription factors are also involved in transcriptional regulation of *OsATG* (autophagy-related) genes under Fe toxicity, and W-box CREs targeted by WRKY TFs are enriched in the promoters of *OsATG* genes (Maltzahn et al., 2020). They suggested these genes are involved in the early Fe toxicity response and may be regulated via WRKY.

### Efficiency of the MAMA Method for Identifying CRE Candidates in the 500 bp Upstream Region

Various approaches to searching for CRE candidates have been described, and it is critical to use an approach that is highly accurate. We hypothesized that genes with similar gene expression patterns that are activated or regulated by the same molecular mechanisms would share the same CRE sets. Thus, in this study, we searched for CRE candidates and confirmed whether the same CREs are shared among genes with the same expression pattern (Figure 7). Searching with the TFBSs which shared among the genes with same expression type, we found that 93% of Fe storage type genes shared the binding sequences of the TBP, AP2, AT-Hook, NF-YB, TCP, homeodomain, B3, bZIP, or alpha-amylase transcription factors (Supplementary Table 2) and up to 90% of genes non-responsive to Fe excess shared the TFBSs of the AT-Hook, NF-YB, TCP, homeodomain, B3, bZIP, SBP, C2H2, or bHLH TFs (Supplementary Table 3). The results were similar between Fe storage type genes and no-response genes, with high percentages of these TFBSs in no-response genes. This condition caused difficulty identifying functional CREs involved in the Fe excess response. This might be because the target area (−1,000 bp from the TSS) was too long to search for frequently occurred CRE sequences such as IDEF1 (5 bp, more than 90% chance of occurrence) and the WRKY binding site. These short and frequently occurring CREs are common in the 1,000 bp upstream sequences of genes. On the other hand, the IDEF1 binding site is enriched in the shorter 500 bp sequence upstream of the TSS of Fe deficiency-regulated genes (Kobayashi et al., 2009).

Thus, the presence of CREs in Fe excess-responsive genes was investigated in the sequences 500 bp upstream of the TSS using reported Fe homeostasis-related CREs (i.e., the binding sequences of IDEF1, IDEF2, IRO2, and WRKY and the IDS1, IDS2, and IDRS promoters) and then we constructed a tree model (Figure 2). CRE prediction using a tree model constructed with XGBoost version 3 and confusion matrix of simulated gene expression patterns did not provide clear results (Figures 3A,B). However, we obtained the highest accuracy of gene expression pattern prediction after using MAMA CRE candidates. The Fe excess response in all tissues was explained by MAMA-predicted CRE candidates with an accuracy of more than 85% (Table 2). Furthermore, in combination with Boruta-XGBoost machine learning methods, these CRE candidates explained a total 80.9% of gene expression patterns correctly (Table 1), showing good consistency with simulated gene expression patterns (Figure 3C). Moreover, a tree model based on a combination of MAMA CREs and PLACE CREs accurately explained 83.0% of gene expression patterns (Figure 3D and Table 1). Our results suggest that MAMA is a powerful tool for CRE prediction to search novel CREs and new role of reported CREs involved in gene regulation of specific tissue/condition/treatment. MAMA can be applied to the transcriptome data such as microarray, RNA-seq, etc. It can also be applied to other living things such as Arabidopsis, mice, humans, etc. as long as the genome database is available.

### Method, Database and Target Sequences of Motif Search

PlantPAN3 uses 30,000 reliable promoters (−5,000 to +1,000 relative to TSSs) and the cut-off of false-positive rate to find motifs was estimated from them. In the following analysis, we compared motif occurrences between regulon and no-response genes and used their shorter promoter regions (−500 to +150 relative to TSSs). As Ksouri et al. (2021) reported, shorter sequence around TSS is much effective for a modeling of CRE. This may explain the difference in results.

CisDB is another largest database of TFBS not only for plants (Weirauch et al., 2014). We attempted to build a Boruta-xgboost model using motifs recorded in CisDB. The highest accuracy using CisDB motifs was 61% (Supplementary Data 1). We searched motifs recorded as position weight matrix (PWM) against background A:T:G:C occurrence in −500 to +150 relative to TSS as {“A”:0.52, “C”: 0.48, “G”: 0.48, “T”: 0.52} (The source code is available at https://github.com/zebul6/Modeling-of-Fe-excess-regulated-transcriptions/tree/main, and Supplementary Data 2–4). Since A:C:G:T occurrence has a position-specific trend around TSS, separating background models to the narrower windows may lead to better accuracy.

We assumed that motifs in PLACE, PlantPAN3, and CisDB are important. It is natural that MAMA scored good because MAMA extracted enriched motifs from regulon. We suggest that CRE prediction methods such as MAMA can provide additional candidates.

### Identifying Novel and Known CRE Candidates Involved in the Fe Excess Response

Both Fe-deficiency stress and Fe excess stress depend on the level of Fe within the rice plant body. Thus, they may be regulated by some of the same mechanisms. For example, ferritin expression is clearly suppressed under Fe-deficiency relative to normal conditions (Ogo et al., 2006), but is strongly induced under Fe excess compared to normal conditions (Aung et al., 2018b). Therefore, we assumed that the expression pattern of the Fe excess response could be better visualized by considering the changes under Fe deficiency treatment reported by Ogo et al. (2006). Furthermore, the behavior of Fe and Zn nutrition in rice and the regulation of gene expression are closely related (Suzuki et al., 2012; Masuda et al., 2020), with OsHRZs and IDEF1 regulating downstream genes by sensing the balance between the availability of Fe and Zn (Kobayashi et al., 2013). Therefore, through comparison of transcriptional response data from studies conducted under Fe excess (Aung et al., 2018b), Fe deficiency (Ogo et al., 2006), and Zn deficiency (Suzuki et al., 2012), we identified 560 CRE candidates (also known as motifs or conserved sequences) related to Fe excess in roots, DC, stems, old leaves, and newest leaf tissues (Supplementary Tables 4–10), in Fe-deficient roots (Supplementary Table 9), and in Zn-deficient roots (Supplementary Table 10) using a high-accuracy CRE prediction tool, the MAMA method. Among these candidates, important motifs were analyzed (Table 3) and their distributions in the upstream sequences of Fe excess-responsive genes were reported (Figures 4, 5 and Supplementary Figures 2–6). The top 10 motifs included known CREs, such as IDEF1 binding (CATGCATG), DCEp2 (ATCGATCG), EECCRCAH1 (PLACE CRE) (GANTTNC), CRTDREHVCBF2 (PLACE CRE) (GTCGAC), and bZIP/bHLH binding (CTACGTGC) motifs. They may play important roles in the Fe excess response and its regulation. IDFE1 is involved in regulatory systems related to both Fe sufficiency and Fe deficiency (Kobayashi et al., 2010). Moreover, we explored new CREs with no previous reports (novel), namely, ATAATGGC, GCWGCWGC, CGACACGC and Myb binding sequence-like (CACCAACC) (Figure 5). These novel CREs might be the binding sequences of important transcription factors that regulate Fe homeostasis in rice.

### Representative Molecular Model Regulating Fe Excess-Responsive Gene Expression Patterns

We generated a representative molecular model for regulation of Fe excess-responsive gene expression patterns using a simple tree model (Figure 7A). Based on these data, we identified specific CRE combinations that commonly occur in each of four gene expression types. We built a model to explain Fe excess-responsive transcriptional regulation based on the combination of presence and absence of CREs predicted with MAMA and PLACE as well as known CREs (Figure 3C). The highest accuracy of the model was 82% (Table 1). The tree model (Figure 7) explained 64% of Fe excess regulated gene expression patterns and was helpful for determining a hypothetical model of transcriptional regulation under Fe excess conditions. Basic core promoters such as the TBP/MADS-box binding (CTATAAAT) and the CpG islands/E2F binding motif (GCGCGCCA) were common in the upstream sequences of Fe storage type genes (Figure 6 and Supplementary Tables 4–8). These promoter sequences are conserved in the upstream sequences of stress-regulated (TATA box) and tissue-specific (CpG islands) genes, and are not common upstream of housekeeping genes (Deaton and Bird, 2011). Stress response and tissue-specific response processes are important to the Fe excess stress response. Therefore, the presence of these CREs upstream of Fe excess genes is reasonable.

A *CAB2* promoter (CAAAACGC) binding site in the dark response element (DtRE) of the chlorophyll a/b-binding protein 2 (CAB2) gene in *Arabidopsis* was commonly shared among metal chelator, WRKY and other co-expression (Figure 7A). These genes are responsive in tissues other than the root (Figure 2). The *CAB2* promoter plays a role in the light response and is a regulator of photosynthesis (Maxwell et al., 2003). Therefore, enrichment of *CAB2* promoter is related to tissue-specific regulation of genes between shoots and roots. A large portion of the gene expression patterns observed in this study were explained by these core promoters and tissue-specific CREs, as regulation in the root is a key factor used to distinguish expression patterns in this study. The FAM1 (AGCTAGCT) and IDEF1 binding sequence (CATGCATG) motifs are common upstream of Fe excess-, Fe- deficiency-, and Zn-deficiency-regulated genes. IDEF1 binding sequence (CATGCATG) motif was important, but FAM1 (AGCTAGCT) was not designated as important (Table 3). A novel candidate CRE motif (CGACACGC) was specifically enriched upstream of Fe excess-regulated genes but not Fe- and Zn-deficiency-regulated genes (Supplementary Tables 9, 10). A Zn-deficiency-related motif (ATAATGGC) was specifically enriched downstream of Zn-deficiency-regulated genes (Figures 5A,B).

The presence of the IDRS core promoter sequence (CCTCCAC) could not be used to explain the transcriptional response to Fe excess, as IDRS was not specifically conserved in the upstream sequences of Fe excess-responsive genes (Figure 3A). However, as Petit et al. (2001) noted, the 3′ sequence of IDRS is important for the Fe excess response. The 3′ sequence of IDRS through GCG may include part of the bHLH binding sequence (G box, CACGTG), although another G box sequence is present in the upstream sequence of *ZmFer1*, and deletion of a G box did not alter the Fe excess response. Overall, the abundance of G box-containing motifs was higher in Fe excess-responsive genes (data not shown, based on Supplementary Tables 4–8). The position of the G box sequence and interactions with the other CREs may affect the G box-regulated response to Fe excess.

In addition to the basic promoter motifs of TATA box, DCEp1, DCEp2 (These three were tandemly co-exist in many genes), E2F binding and CpG islands, motifs such as the IDEF1 binding sequence-containing motif CATGCATG and FAM1, were commonly enriched among genes responsive to Fe excess, Fe deficiency, and Zn deficiency (Supplementary Tables 4–10), and therefore were selected as important motifs for explaining gene expression patterns in the Fe response. Our findings suggest that Fe deficiency, Zn deficiency, and Fe excess may partially share the same regulatory mechanism. Nowadays, many reports have noted that Fe deficiency and Fe excess are regulated by the same or similar transcription factors. Among *AtbHLH* genes, *AtbHLH104* and *AtbHLH034* are involved in regulation under Fe deficiency, whereas *AtbHLH047* (PYE) is involved in Fe excess (Zhang et al., 2015; Li et al., 2016; Liang et al., 2017). *OsHRZ* is related to regulation under both Fe deficiency and Fe excess conditions (Kobayashi et al., 2013; Aung et al., 2018a).

There are previous studies of identification of the important CREs which were able to use as a powerful promoter. Kobayashi et al. (2003) identified functional two CREs, IDE1 and IDE2 (iron-deficiency-responsive element 1 and 2), which synergistically induced Fe-deficiency-specific expression in tobacco roots. Using those CREs, they constructed artificial promoters that highly respond to Fe deficiency in rice (Kobayashi et al., 2004). Furthermore, Hayami et al. (2015) also reported that based on new CREs related to stress response (cold, high light, and UV-B) explored by computer simulation, they could artificially produce new promoters which lead to a high expression under stress condition (cold, high light, and UV-B). In this study, we only selected the best motifs to explain Fe excess-responsive expression patterns. Further studies are required to confirm that these motifs function *in vivo*. Then, it is possible to produce powerful Fe excess-responsive promoters and subsequently to produce a new Fe excess tolerant rice by enhancing the expression of target genes using those artificial promoters. Moreover, based on the novel CREs sequences related to the Fe excess response identified in this study, new transcription factors that bind to these CREs and regulate the rice Fe excess response through unknown mechanisms might be found in the future.

## Conclusion

We observed four gene expression types in the Fe excess response through gene network analyses based on microarray data collected under Fe excess, Fe deficiency, and Zn deficiency. We elucidated a total of 560 CREs (also known as motifs or conserved sequences) directly related to Fe excess response mechanism in various rice tissues using machine learning approaches. Here, we report novel CREs as well as known CREs that were significantly related to the Fe excess response. Moreover, we developed a model regulating Fe excess-responsive genes based on the identified *cis*-elements. Overall, our results provide novel CREs and conserved sequences that may be used as an important data source for further clarification of the Fe excess response mechanism in rice, discovery of genes and transcription factors involved in Fe excess-responsive pathways. In addition, these novel CRE sequences represent an important source of information for studies aimed at modifying promoter sequences or enhancing gene expression to produce new rice varieties that are tolerant of Fe excess.

## Data Availability Statement

The original contributions presented in the study are included in the article/Supplementary Material, further inquiries can be directed to the corresponding author/s.

## Author Contributions

YK, MSA, and HM conceived of the study and methodology. MSA, HM, and NKN provided microarray data. YK analyzed and prepared the data. YK, HM, and MSA led the writing of the manuscript, with suggestions from NKN and HH. MSA, NKN, and HH supported the project. All authors interpreted the data, contributed to the article, and approved the submitted version.

## Conflict of Interest

The authors declare that the research was conducted in the absence of any commercial or financial relationships that could be construed as a potential conflict of interest.

## Footnotes

1 https://rapdb.dna.affrc.go.jp/download/irgsp1.html
