# Supplementary material for: Elucidation of Novel cis-Regulatory Elements and Promoter Structures Involved in Iron Excess Response Mechanisms in Rice Using a Bioinformatics Approach
Source: Front Plant Sci. 2021 Jun 2;12:660303. doi: 10.3389/fpls.2021.660303 (PMC8207140; doi:10.3389/fpls.2021.660303)
Supplement: Supplementary file 1 [file Data_Sheet_1.zip › Supplementary Data 2.docx]

**Supplementary Data 2**

##################################################

## Code for WGCNA analysis (Kakei et al. 2021)

##################################################

## Two color Agilent microarray data (Fe excess) were preprocessed by limma package uging following code.

#x <- read.maimages(targets, path="./", source="agilent",green.only=FALSE)

#y <- backgroundCorrect(x, method="normexp", offset=16)

#y <- normalizeWithinArrays(y, method="robustspline") #Two coloarrayr

#y <- normalizeBetweenArrays(y, method="quantile")

## Log 2 gene expression ratio was recorded in exptableM.txt

#tmp=cbind(y$genes$ProbeName, y$genes$SystematicName, y$M)

#colnames(tmp) <- c("ProbeName", "SystematicName", as.vector(y$targets$Cy5))

#write.table(tmp, file="exptableM.txt", sep="\t", quote=FALSE)

## The geometric mean of gene expression ratios (exptableM.txt) of the Fe-excess groups for each tissue were used for analyses because clustering analyses of the microarray data showed that gene expression patterns under Fe excess ratios of ×10, ×20, ×50, and ×70 were generally consistent (Supplementary Figure 1).

## In addition, microarray data on Fe-deficient rice roots were obtained from Ogo et al. (2006). Microarray data on Zn-deficient rice roots were obtained from Suzuki et al. (2012).

## The log2 gene expression data of regulon were recorded in SelectRepresentativeREG.csv

## LocusID and gene Symbol (Geme name) were added using IRGSP-1.0 to the file.

data <- read.csv("SelectRepresentativeREG.csv") #Contact us if you need this.

rownames(data) <- data$Symbol

rownames(data) <- data$LocusID

if (!requireNamespace("BiocManager", quietly = TRUE))

install.packages("BiocManager")

BiocManager::install("WGCNA")

library(WGCNA)

## Generation of network graph using WGCNA from exptableM.txt

## Following Tutorial for the GGCNA package for R (Peter Langfelder and Steve Horvath, 2014)

options(stringsAsFactors = FALSE)

enableWGCNAThreads()

datExpr0 = as.data.frame(data[,5:dim(data)[2]]) # ProbeName, LocusID and SystematicName were removed.

gsg = goodSamplesGenes(datExpr0, verbose = 3);

gsg$allOK

if (!gsg$allOK)

{

# Optionally, print the gene and sample names that were removed:

if (sum(!gsg$goodGenes)>0)

printFlush(paste("Removing genes:", paste(names(datExpr0)[!gsg$goodGenes], collapse = ", ")));

if (sum(!gsg$goodSamples)>0)

printFlush(paste("Removing samples:", paste(rownames(datExpr0)[!gsg$goodSamples], collapse = ", ")));

# Remove the offending genes and samples from the data:

datExpr0 = datExpr0[gsg$goodSamples, gsg$goodGenes]

}

library(flashClust)

sampleTree = flashClust(dist(datExpr0), method = "average");

# Plot the sample tree: Open a graphic output window of size 12 by 9 inches

# The user should change the dimensions if the window is too large or too small.

#sizeGrWindow(12,9)

pdf(file = "200427_GCWNA_GenesClustering.pdf", width = 12, height = 9);

par(cex = 0.6);

par(mar = c(0,4,2,0))

plot(sampleTree, main = "Sample clustering to detect outliers", sub="", xlab="", cex.lab = 1.5,

cex.axis = 1.5, cex.main = 2)

# Plot a line to show the cut

abline(h = 5, col = "red");

# Determine cluster under the line

clust = cutreeStatic(sampleTree, cutHeight = 5, minSize = 2)

table(clust)

# clust 1 contains the samples we want to keep.

#keepSamples = (clust==1)

#datExpr = as.data.frame(t(datExpr0[keepSamples, ]))

# We kept all the genes because we already selected significantly regulated regulons.

datExpr = as.data.frame(t(datExpr0))

nGenes = ncol(datExpr)

nSamples = nrow(datExpr)

dev.off()

save(datExpr, file = "01-dataInput.RData")

clusterResult <- rbind(datExpr, clust)

write.table(clusterResult, file="200427_GeneClusters.txt")

# Load the data saved in the first part

#lnames = load(file = "01-dataInput.RData");

#The variable lnames contains the names of loaded variables.

#lnames

# Choose a set of soft-thresholding powers

powers = c(c(1:10), seq(from = 12, to=20, by=2))

# Call the network topology analysis function

sft = pickSoftThreshold(datExpr, powerVector = powers, verbose = 5)

# Plot the results:

sizeGrWindow(9, 5)

par(mfrow = c(1,2));

cex1 = 0.9;

# Scale-free topology fit index as a function of the soft-thresholding power

plot(sft$fitIndices[,1], -sign(sft$fitIndices[,3])*sft$fitIndices[,2],

xlab="Soft Threshold (power)",ylab="Scale Free Topology Model Fit,signed R^2",type="n",

main = paste("Scale independence"));

text(sft$fitIndices[,1], -sign(sft$fitIndices[,3])*sft$fitIndices[,2],

labels=powers,cex=cex1,col="red");

# this line corresponds to using an R^2 cut-off of h

abline(h=0.90,col="red")

# Mean connectivity as a function of the soft-thresholding power

plot(sft$fitIndices[,1], sft$fitIndices[,5],

xlab="Soft Threshold (power)",ylab="Mean Connectivity", type="n",

main = paste("Mean connectivity"))

text(sft$fitIndices[,1], sft$fitIndices[,5], labels=powers, cex=cex1,col="red")

net = blockwiseModules(datExpr, power = 6,

TOMType = "unsigned", minModuleSize = 3,

reassignThreshold = 1, mergeCutHeight = 0.2,

numericLabels = TRUE, pamRespectsDendro = FALSE,

saveTOMs = TRUE,

saveTOMFileBase = "TOM",

verbose = 5)

# open a graphics window

sizeGrWindow(12, 9)

# Convert labels to colors for plotting

mergedColors = labels2colors(net$colors)

# Plot the dendrogram and the module colors underneath

plotDendroAndColors(net$dendrograms[[1]], mergedColors[net$blockGenes[[1]]],

"Module colors",

dendroLabels = FALSE, hang = 0.02,

addGuide = TRUE, guideHang = 0.05)

moduleLabels = net$colors

moduleColors = labels2colors(net$colors)

MEs = net$MEs;

geneTree = net$dendrograms[[1]];

save(MEs, moduleLabels, moduleColors, geneTree,

file = "02-networkConstruction-auto.RData")

# Load network data saved in the second part.

lnames = load(file = "02-networkConstruction-auto.RData");

lnames

nGenes = ncol(datExpr)

nSamples = nrow(datExpr)

# Calculate topological overlap anew: this could be done more efficiently by saving the TOM

# calculated during module detection, but let us do it again here.

dissTOM = 1-TOMsimilarityFromExpr(datExpr, power = 6);

# Transform dissTOM with a power to make moderately strong connections more visible in the heatmap

plotTOM = dissTOM^7;

# Set diagonal to NA for a nicer plot

diag(plotTOM) = NA;

# Call the plot function

sizeGrWindow(9,9)

TOMplot(plotTOM, geneTree, moduleColors, main = "Network heatmap plot, all genes")

dev.off()

# Recalculate topological overlap if needed

TOM = TOMsimilarityFromExpr(datExpr, corType = "pearson", power = 6);

# Select modules

modules = c("grey", "turquoise", "blue", "brown", "yellow", "green");

# Select module probes

probes = names(datExpr)

inModule = is.finite(match(moduleColors, modules));

modProbes = probes[inModule];

# Select the corresponding Topological Overlap

modTOM = TOM[inModule, inModule];

dimnames(modTOM) = list(modProbes, modProbes)

# Export the network into edge and node list files Cytoscape can read

cyt = exportNetworkToCytoscape(modTOM,

edgeFile = paste("CytoscapeInput-edges-", ".txt", sep=""),

nodeFile = paste("CytoscapeInput-nodes-", ".txt", sep=""), weighted = TRUE, threshold = 0.1, nodeNames = modProbes, nodeAttr = moduleColors[inModule]);

#install.packages("igraph")

library(igraph)

infile <- paste("CytoscapeInput-edges-", ".txt", sep="")

d <- read.table(infile, sep="\t",header=T)

library(data.table)

d <- fread(infile)

g <- graph.data.frame(d[2:nrow(d),1:2],directed=F)

#E(g)$weight <- d[3]

g <- simplify(g,remove.multiple=F,remove.loops=T)

E(g)$width=0.3

E(g)$color="gray"

V(g)$size <- 2

V(g)$label.cex = 0.7

V(g)$frame.color = "white"

mst <- minimum.spanning.tree(g)

#l <- layout.reingold.tilford(g, mode="all")

#l <- layout.reingold.tilford(g, circular=T)

l <- layout.kamada.kawai(g)

pdf(file="network_outfile_WGCNA-all-00001-1.pdf",width=16,height=12,fonts=c("serif"))

plot(g,layout=l,vertex.label.color="black",edge.arrow.size=0.3)

#plot(g,layout=l,vertex.label="",edge.arrow.size=0.3)

dev.off()

l <- layout.fruchterman.reingold(g,niter=1000,area=5*vcount(g)^2,repulserad=vcount(g)^2.8)

pdf(file="network_outfile_WGCNA-all-00001-2.pdf",width=16,height=12,fonts=c("serif"))

plot(g,layout=l,vertex.label.color="black",edge.arrow.size=0.3)

#plot(g,layout=l,vertex.label="",edge.arrow.size=0.3)

dev.off()

## leyout by fruchterman.reingold looks better to understand clustering result
