# Supplementary material for: Elucidation of Novel cis-Regulatory Elements and Promoter Structures Involved in Iron Excess Response Mechanisms in Rice Using a Bioinformatics Approach
Source: Front Plant Sci. 2021 Jun 2;12:660303. doi: 10.3389/fpls.2021.660303 (PMC8207140; doi:10.3389/fpls.2021.660303)
Supplement: Supplementary file 1 [file Data_Sheet_1.zip › Supplementary Data 3.docx]

**Supplementary Data 3**

#########################################################

# Code for MAMA-Boruta modeling of gene expression (Kakei et al. 2021)

#########################################################

library(xgboost)

library(Boruta)

require(rpart)

require(RColorBrewer)

library(ROCR)

library(caret)

library(dplyr)

library(rattle)

library(readr)

## Preparation for parallel calculation

library(parallel)

no_cores <- detectCores() - 1

library(doParallel)

cl <- makePSOCKcluster(no_cores)

registerDoParallel(cl)

## Function to estimate the best subsumpling rate to divide training:test data

# and number of cross varivation in train data.

estimate_data_split <- function(data,niter){

best_accuracy = 0

best_sampling_rate = 0

for (iter in c(1:niter)){

seed.number = sample.int(10000, 1)[[1]]

set.seed(seed.number)

sampling_rate = sample(seq(from = 2, to = 5, by = 0.2),size=1)

num_cv = sample(seq(from = 2, to = 5, by = 1),size=1)

inTest <- sample(c(1:(dim(data)[1])), floor(dim(data)[1] * (1/sampling_rate)))

colsort <- sample(c(2:(dim(data)[2])), floor(dim(data)[2]-1 ))

train<- data[-inTest, c(1,colsort)]

test <- data[inTest, c(1,colsort)]

train_x = data.matrix(train[,-1])

train_y = train$y

test_x = data.matrix(test[,-1])

test_y = test$y

down_test <- downSample(x = test_x,

y = test$y)

tune_grid <- expand.grid(

nrounds = seq(from = 200, to = 1000, by = 200),

eta = c(0.025, 0.05, 0.1),

max_depth = c(2, 3, 4, 5, 6),

gamma = 0,

colsample_bytree = 1,

min_child_weight = 1,

subsample = 1

)

tune_control <- caret::trainControl(

method = "cv", #"repeatedcv",

number = num_cv, # c(2, 3, 4, 5, 10)

#repeats = 5,

#index = createFolds(tr_treated$Id_clean), # fix the folds

verboseIter = FALSE, # no training log

allowParallel = TRUE, # FALSE for reproducible results, TRUE for speed

sampling = "down" # c("up","down","smote")

)

xgb_base <- caret::train(

x= train_x,

y = train_y,

trControl = tune_control,

tuneGrid = tune_grid,

method = "xgbTree",

verbose = TRUE

)

pred = predict(xgb_base, down_test[,-dim(down_test)[2]])

accur = mean(as.data.frame(caret::confusionMatrix(pred , down_test$Class)$byClass)[,11])

# accur = caret::confusionMatrix(pred, down_test$Class)$overall["Accuracy"]

# Balanced accuracy (Average of Specificity and Sensitivity) was used because data is imbalanced.

if(accur > best_accuracy){

best_accuracy <- accur

best_sampling_rate <- sampling_rate

best_inTest <- inTest

best_colsort <- colsort

best_seednumber <- seed.number

xgb_best <- xgb_base

best_test <- test

best_train <- train

best_down_test <- down_test

}

}

#best_sampling_rate # 4

#best_accuracy #0.63

#write(best_colsort,file="best_colsort")

#best_seednumber #4038

return(list(

best_sampling_rate=best_sampling_rate,

best_accuracy=best_accuracy,

best_seednumber=best_seednumber,

xgb_best = xgb_best,

best_test = test,

best_train = train,

best_down_test = best_down_test

))

}

## Function to reproduce the best model

reproduce_bestmodel <- function(data,best_seednumber){

set.seed(best_seednumber)

sampling_rate = sample(seq(from = 2, to = 5, by = 0.2),size=1)

num_cv = sample(seq(from = 2, to = 5, by = 1),size=1)

inTest <- sample(c(1:(dim(data)[1])), floor(dim(data)[1] * (1/sampling_rate)))

colsort <- sample(c(2:(dim(data)[2])), floor(dim(data)[2]-1 ))

train<- data[-inTest, c(1,colsort)]

test <- data[inTest, c(1,colsort)]

train_x = data.matrix(train[,-1])

train_y = train$y

test_x = data.matrix(test[,-1])

test_y = test$y

tune_grid <- expand.grid(

nrounds = seq(from = 200, to = 1000, by = 200),

eta = c(0.025, 0.05, 0.1),

max_depth = c(2, 3, 4, 5, 6),

gamma = 0,

colsample_bytree = 1,

min_child_weight = 1,

subsample = 1

)

tune_control <- caret::trainControl(

method = "cv",

number = num_cv,

verboseIter = FALSE, # no training log

allowParallel = FALSE, # FALSE for reproducible results, TRUE for speed

sampling = "down" # c("up","down","smote")

)

xgb_base <- caret::train(

x= train_x,

y = train_y,

trControl = tune_control,

tuneGrid = tune_grid,

method = "xgbTree",

verbose = TRUE

)

return(xgb_base)

}

## Function to reproduce the best split

reproduce_split <- function(data,best_seednumber){

set.seed(best_seednumber)

sampling_rate = sample(seq(from = 2, to = 5, by = 0.2),size=1)

num_cv = sample(seq(from = 2, to = 5, by = 1),size=1)

inTest <- sample(c(1:(dim(data)[1])), floor(dim(data)[1] * (1/sampling_rate)))

colsort <- sample(c(2:(dim(data)[2])), floor(dim(data)[2]-1 ))

train<- data[-inTest, c(1,colsort)]

test <- data[inTest, c(1,colsort)]

return(list(

train=train,

test=test,

colsort=colsort

))

}

########################################################

# Consctuction of MAMA-Boruta model

########################################################

## Clustered genes with clustername and promoter sequence (-500 to +150) are read

# This file includes unnamed genes and genes not responded in addition to the genes in Fig. 2

# colnames = GeneID, sequence , clustered-category

data <- read_csv("../200623ModelingClusteredUnnamedNoResponse650.csv",col_names = FALSE)

colnames(data)[3] <- "y"

class <- data$y

## Loading of Search results of MAMA extracted motifs

data1 <- readr::read_csv("./motifcount_IronDef.csv")

data1 <- data1[,-dim(data1)[2]]

data2 <- read_csv("./motifcount_ZincDef.csv")

data2 <- data2[,-dim(data2)[2]]

data3 <- read_csv("./motifcount_Newest_leaf.csv")

data3 <- data3[,-dim(data3)[2]]

data4 <- read_csv("./motifcount_Old_leaf.csv")

data4 <- data4[,-dim(data4)[2]]

data5 <- read_csv("./motifcount_Root.csv")

data5 <- data5[,-dim(data5)[2]]

data6 <- read_csv("./motifcount_Stem.csv")

data6 <- data6[,-dim(data6)[2]]

data7 <- read_csv("./motifcount_DC.csv")

data7 <- data7[,-dim(data7)[2]]

cols_remove <- c(1,2) # delete GeneID and sequence

## Order of data here affects to the modeling as well as seed numbers

data <- bind_cols(data4[,-1],data5[,-cols_remove],data1[,-cols_remove],data2[,-cols_remove],data3[,-cols_remove],data6[,-cols_remove],data7[,-cols_remove])

colnames(data)[1] <- "y"

data$y <- as.factor(class)

indx <- 2:length(colnames(data))

data[indx] <- lapply(data[indx], function(x) as.integer(as.factor(x))) # convert tha data to 2(present)/1(absent)

## Construction and test of simulation model

set.seed(1500)

best_split <- estimate_data_split(data,300)

pred = predict(best_split$xgb_best, best_split$best_down_test[,-dim(best_split$best_down_test)[2]])

caret::confusionMatrix(pred , best_split$best_down_test$Class)

accur_in_test = mean(as.data.frame(caret::confusionMatrix(pred , best_split$best_down_test$Class)$byClass)[,11])

# 62.5% Accuracy

datasplit <- reproduce_split(data,best_split$best_seednumber)

pred = predict(best_split$xgb_best, data.matrix(data[,datasplit$colsort]))

accur_in_alldata = mean(as.data.frame(caret::confusionMatrix(pred, data$y)$byClass)[,11])

# 82% Accuracy

# Save the result of statistical analysis

sink("20210316_stat_MAMA_model.txt")

print(caret::confusionMatrix(pred, data$y))

sink()

## Application of boruta feature selection method

# Boruta selects better motif than random presense/absense data

set.seed(2869)

Boruta(y~.,data=data,

maxRuns = 500,doTrace=2)->Bor.son

sink("boruta_mama_result.txt")

print(Bor.son)

sink()

stats<-attStats(Bor.son)

sink("boruta_mama_stats.txt")

print(stats)

sink()

pdf(file="boruta_mama_plot.pdf")

plot(normHits~meanImp,col=stats$decision,data=stats)

dev.off()

## Select "Confirmed" motifs by Boruta

selected_boruta <- rownames(attStats(Bor.son))[which(attStats(Bor.son)$decision == "Confirmed")]

borutadata <- select(.data = data, all_of(c("y", selected_boruta)) )

## Construction and test of simulation model

best_split <- estimate_data_split(borutadata,300)

pred = predict(best_split$xgb_best, best_split$best_down_test[,-dim(best_split$best_down_test)[2]])

caret::confusionMatrix(pred , best_split$best_down_test$Class)

accur_in_test = mean(as.data.frame(caret::confusionMatrix(pred , best_split$best_down_test$Class)$byClass)[,11])

# 67%

datasplit <- reproduce_split(borutadata,best_split$best_seednumber)

pred = predict(best_split$xgb_best, data.matrix(borutadata[,datasplit$colsort]))

accur_in_alldata = mean(as.data.frame(caret::confusionMatrix(pred, borutadata$y)$byClass)[,11])

# 80.6%

# Save the result of statistical analysis

caret::confusionMatrix(pred, borutadata$y)

sink("20210316_stat_MAMA-Boruta_model650.txt")

print(caret::confusionMatrix(pred, data$y))

sink()

########################################################

# Consctuction of PLACE-Boruta model

########################################################

# Preprocess to reand prepare motif count of PLACE cis-regulatory elements

placedata <- read_csv("./motifcount_placeCRE.csv")

colnames(placedata)[2] <- "y"

placedata$y <- as.factor(class)

placedata <- placedata[,-1]

placedata <- placedata[,-dim(placedata)[2]]

intplacedata <- placedata

indx <- 2:length(colnames(placedata))

intplacedata[,indx] <- lapply(placedata[indx], function(x) as.integer(as.factor(x))) # convert tha data to 2(present)/1(absent)

# Deletion of motifs that does not exist on promoters exist on all promoters

intplacedata <- intplacedata[,-which(lapply(intplacedata, function(x) length(levels(as.factor(x)))) == 1)]

# Motif names that start with numbers cannot be applied for Boruta modeling

colnames(intplacedata) <- unlist(lapply(colnames(intplacedata), function(x) sprintf("X_%s",x)))

colnames(intplacedata)[1] <- "y" #revert from "X_Y"

## Application of boruta feature selection method

set.seed(4598)

Boruta(y~.,data=intplacedata,

maxRuns = 300,doTrace=2)->Bor.place

selected_place <- rownames(attStats(Bor.place))[which(attStats(Bor.place)$decision == "Confirmed")]

borutaplaceintdata <- select(.data = intplacedata, all_of(c("y", selected_place)))

## Construction and test model

best_split <- estimate_data_split(borutaplaceintdata,300)

pred = predict(best_split$xgb_best, best_split$best_down_test[,-dim(best_split$best_down_test)[2]])

caret::confusionMatrix(pred , best_split$best_down_test$Class)

accur_in_test = mean(as.data.frame(caret::confusionMatrix(pred , best_split$best_down_test$Class)$byClass)[,11])

# 62.5% Accuracy

datasplit <- reproduce_split(borutaplaceintdata,best_split$best_seednumber)

pred = predict(best_split$xgb_best, data.matrix(borutaplaceintdata[,datasplit$colsort]))

accur_in_alldata = mean(as.data.frame(caret::confusionMatrix(pred, borutaplaceintdata$y)$byClass)[,11])

# 63% Accuracy

# Save the result of statistical analysis

sink("20210316_stat_PLACE-Boruta_model.txt")

print(caret::confusionMatrix(pred, borutaplaceintdata$y))

sink()

# 67%

########################################################

# Consctuction of PLACE&MAMA-Boruta model

########################################################

# Integration of PLACE and MAMA selected motifs

intplacemamadata <- bind_cols(borutadata,borutaplaceintdata[,-1])

## Construction and test model

best_split <- estimate_data_split(intplacemamadata,300)

pred = predict(best_split$xgb_best, best_split$best_down_test[,-dim(best_split$best_down_test)[2]])

caret::confusionMatrix(pred , best_split$best_down_test$Class)

accur_in_test = mean(as.data.frame(caret::confusionMatrix(pred , best_split$best_down_test$Class)$byClass)[,11])

# 63% Accuracy

datasplit <- reproduce_split(intplacemamadata,best_split$best_seednumber)

pred = predict(best_split$xgb_best, data.matrix(intplacemamadata[,datasplit$colsort]))

accur_in_alldata = mean(as.data.frame(caret::confusionMatrix(pred, intplacemamadata$y)$byClass)[,11])

# 83.0% Accuracy

# Save the result of statistical analysis

sink("20210316_stat_PLACEandMAMA-Boruta_model650.txt")

print(caret::confusionMatrix(pred, intplacemamadata$y))

sink()

# Save importance of motifs in the best model

sink("20210316_importance_PLACEandMAMA-Boruta_model650.txt")

print(varImp(best_split$xgb_best))

sink()

## Construction of Simple Tree model by party package

formula = as.formula(y ~.)

library(tcltk)

maxtry = 10000

best_acc=0

pb <- txtProgressBar(min = 1, max = maxtry, style = 3)

for (b in 1:maxtry){

seed.number = sample.int(n=10000, 1)

set.seed(seed.number)

rpart.Control <- trainControl(method = "cv", # use N-fold cross validation

number = sample(2:5,1), # the number of folds

classProbs = TRUE,

summaryFunction = multiClassSummary,

sampling=sample(c("down","smote"),1))

colsort = sample((1:dim(intplacemamadata)[2]),dim(intplacemamadata)[2])

t.AUC <- train(formula,intplacemamadata[,colsort], method = "rpart", trControl = rpart.Control, metric = "AUC", tuneGrid = data.frame(cp=c(0.05,0.02,0.002,0.0002)))

x <- capture.output(print(t.AUC$finalModel))

acc <- mean(as.data.frame(caret::confusionMatrix(predict(t.AUC), intplacemamadata$y)$byClass)[,11])

if(n > 1 && acc > best_acc){

best.t.AUC <- t.AUC

best_acc <- acc

}

setTxtProgressBar(pb, b)

}

pdf(file="simpletree_down_boruta650.pdf")

#plot(t.AUC) # uncomment to check cp-AUC relations

fancyRpartPlot(best.t.AUC$finalModel)

dev.off()

predict_rpart_model <- predict(best.t.AUC)

mean(as.data.frame(caret::confusionMatrix(predict_rpart_model, intplacemamadata$y)$byClass)[,11])

#ACC 0.73

# Save the result of statistical analysis

sink("20210316_stat_simple_tree_model650.txt")

print(caret::confusionMatrix(predict_rpart_model, intplacemamadata$y))

sink()

names(varImp(best.t.AUC)$importance)

rownames(varImp(t.AUC)$importance)[which(varImp(t.AUC)$importance > 0)]

sink("20210316_importance_simple_tree_model650.txt")

varImp(best.t.AUC)

sink()

#rpart variable importance

#

# only 20 most important variables shown (out of 30)

#

# Overall

#CATGCATG 100.00

#CATCACAC 95.62

#AGCTAAGC 80.13

#GTACACCT 61.24

#CGAGCGCG 43.14

#CTCGCTAG 42.00

#GTGATCAC 38.18

#CGACACGC 37.50

#X_5659BOXLELAT5659 35.89

#X_OCETYPEIINTHISTONE 34.53

#GCGCGCCA 32.76

#X_ABREBZMRAB28 31.82

#X_SGBFGMGMAUX28 28.37

#CGCGACAC 27.81

#X_ABREOSRAB21 26.34

#AGCTAGCT 25.90

#GCCACACG 19.83

#GATCACCA 18.44

#GCACACGC 13.47

#GCAGCAGC 13.05

########################################################

# Consctuction of Known CIS-Boruta model

########################################################

placedata <- read_csv("./motifcount_placeCRE.csv")

colnames(placedata)[2] <- "y"

placedata$y <- as.factor(class)

placedata <- placedata[,-1]

placedata <- placedata[,-dim(placedata)[2]]

knowncisdata <- placedata[,c(1:9)]

intknowncisdata <- knowncisdata

indx <- 2:length(colnames(knowncisdata))

intknowncisdata[,indx] <- lapply(intknowncisdata[indx], function(x) as.integer(as.factor(x))) # convert tha data to 1(present)/0(absent)

# delete motifs not exist on promoters

intknowncisdata <- intknowncisdata[,-which(lapply(intknowncisdata, function(x) length(levels(as.factor(x)))) == 1)]

## Construction and test model

best_split <- estimate_data_split(intknowncisdata,300)

pred = predict(best_split$xgb_best, best_split$best_down_test[,-dim(best_split$best_down_test)[2]])

caret::confusionMatrix(pred , best_split$best_down_test$Class)

accur_in_test = mean(as.data.frame(caret::confusionMatrix(pred , best_split$best_down_test$Class)$byClass)[,11])

# 58%

datasplit <- reproduce_split(intknowncisdata,best_split$best_seednumber)

pred = predict(best_split$xgb_best, data.matrix(intknowncisdata[,datasplit$colsort]))

accur_in_alldata = mean(as.data.frame(caret::confusionMatrix(pred, intknowncisdata$y)$byClass)[,11])

sink("20210316_stat_knownCIS_model.txt")

print(caret::confusionMatrix(pred, intknowncisdata$y))

sink()

write.table(caret::confusionMatrix(pred, intknowncisdata$y),"20210316_stat_knownCIS_model.txt")

# 56%

########################################################

# Consctuction of CISDB-Boruta model

########################################################

## Load Search results of CIS-DB motifs (by search_pwm.py)

data_cisdb1 <- read_csv("./motifcount_CISBP1.csv")

colnames(data_cisdb1)[1] <- "y"

data_cisdb1$y <- as.factor(class)

indx <- 2:length(colnames(data_cisdb1))

data_cisdb1[indx] <- lapply(data_cisdb1[indx], function(x) as.integer(as.factor(x)))

## boruta feature selection method

# Boruta selects better motif than random presense/absense data

Boruta(y~.,data=data_cisdb1,

maxRuns = 500,doTrace=2)->Bor.son

## Select "Confirmed" motifs by Boruta

selected_boruta <- rownames(attStats(Bor.son))[which(attStats(Bor.son)$decision == "Confirmed")]

#[1] "M02039_2.00.txt" "M00863_2.00.txt" "M01217_2.00.txt" "M05523_2.00.txt"

#[5] "M07291_2.00.txt" "M01768_2.00.txt"

cisdb1data <- select(.data = data_cisdb1, all_of(c("y", selected_boruta)) )

## Construction and test model

best_split <- estimate_data_split(cisdb1data,20)

pred = predict(best_split$xgb_best, best_split$best_down_test[,-dim(best_split$best_down_test)[2]])

caret::confusionMatrix(pred , best_split$best_down_test$Class)

accur_in_test = mean(as.data.frame(caret::confusionMatrix(pred , best_split$best_down_test$Class)$byClass)[,11])

# 61%

datasplit <- reproduce_split(cisdb1data,best_split$best_seednumber)

pred = predict(best_split$xgb_best, data.matrix(cisdb1data[,datasplit$colsort]))

accur_in_alldata = mean(as.data.frame(caret::confusionMatrix(pred, cisdb1data$y)$byClass)[,11])

caret::confusionMatrix(pred, borutadata$y)

sink("20210316_stat_CISDB1-Boruta_model.txt")

print(caret::confusionMatrix(pred, data$y))

sink()

# 58%

## Load Search results of CIS-DB motifs (by search_pwm.py)

data_cisdb2 <- read_csv("./motifcount_CISBP2.csv")

colnames(data_cisdb2)[1] <- "y"

data_cisdb2$y <- as.factor(class)

indx <- 2:length(colnames(data_cisdb2))

data_cisdb2[indx] <- lapply(data_cisdb2[indx], function(x) as.integer(x))

Boruta(y~.,data=data_cisdb2,

maxRuns = 500,doTrace=2)->Bor.son.cisdb2

# Select "Confirmed" motifs by Boruta

selected_boruta <- rownames(attStats(Bor.son.cisdb2))[which(attStats(Bor.son.cisdb2)$decision == "Confirmed")]

cisdb2data <- select(.data = data_cisdb1, all_of(c("y", selected_boruta)) )

## Construction and test model

best_split <- estimate_data_split(cisdb2data,20)

pred = predict(best_split$xgb_best, best_split$best_down_test[,-dim(best_split$best_down_test)[2]])

caret::confusionMatrix(pred , best_split$best_down_test$Class)

accur_in_test = mean(as.data.frame(caret::confusionMatrix(pred , best_split$best_down_test$Class)$byClass)[,11])

# 58%

datasplit <- reproduce_split(cisdb2data,best_split$best_seednumber)

pred = predict(best_split$xgb_best, data.matrix(cisdb2data[,datasplit$colsort]))

accur_in_alldata = mean(as.data.frame(caret::confusionMatrix(pred, cisdb2data$y)$byClass)[,11])

caret::confusionMatrix(pred, borutadata$y)

sink("20210316_stat_CISDB2-Boruta_model.txt")

print(caret::confusionMatrix(pred, data$y))

sink()

# 54%

## Load Search results of CIS-DB motifs (by search_pwm.py)

data_cisdb3 <- read.csv("./motifcount_CISBP3.csv",header=T)

colnames(data_cisdb3)[1] <- "y"

data_cisdb3$y <- as.factor(class)

indx <- 2:length(colnames(data_cisdb3))

data_cisdb3[indx] <- lapply(data_cisdb3[indx], function(x) as.integer(x))

Boruta(y~.,data=data_cisdb3,

maxRuns = 500,doTrace=2)->Bor.son.cisdb3

# Select "Confirmed" motifs by Boruta

selected_boruta <- rownames(attStats(Bor.son.cisdb3))[which(attStats(Bor.son.cisdb3)$decision == "Confirmed")]

cisdb3data <- select(.data = data_cisdb1, all_of(c("y", selected_boruta)) )

## Construction and test model

best_split <- estimate_data_split(cisdb3data,20)

pred = predict(best_split$xgb_best, best_split$best_down_test[,-dim(best_split$best_down_test)[2]])

caret::confusionMatrix(pred , best_split$best_down_test$Class)

accur_in_test = mean(as.data.frame(caret::confusionMatrix(pred , best_split$best_down_test$Class)$byClass)[,11])

# 61%

datasplit <- reproduce_split(cisdb3data,best_split$best_seednumber)

pred = predict(best_split$xgb_best, data.matrix(cisdb3data[,datasplit$colsort]))

accur_in_alldata = mean(as.data.frame(caret::confusionMatrix(pred, cisdb3data$y)$byClass)[,11])

caret::confusionMatrix(pred, borutadata$y)

sink("20210316_stat_CISDB3-Boruta_model.txt")

print(caret::confusionMatrix(pred, data$y))

sink()

# 56%

## Load Search results of CIS-DB motifs (by search_pwm.py)

data_cisdb_fpr001 <- read.csv("./motifcount_CISBP_fpr001.csv",header=T)

colnames(data_cisdb_fpr001)[1] <- "y"

data_cisdb_fpr001$y <- as.factor(class)

indx <- 2:length(colnames(data_cisdb_fpr001))

data_cisdb_fpr001[indx] <- lapply(data_cisdb_fpr001[indx], function(x) as.integer(x))

Boruta(y~.,data=data_cisdb_fpr001,

maxRuns = 500,doTrace=2)->Bor.son.cisdb_fpr001

# Select "Confirmed" motifs by Boruta

selected_boruta <- rownames(attStats(Bor.son.cisdb_fpr001))[which(attStats(Bor.son.cisdb_fpr001)$decision == "Confirmed")]

cisdb_fpr001data <- select(.data = data_cisdb1, all_of(c("y", selected_boruta)) )

## Construction and test model

best_split <- estimate_data_split(cisdb_fpr001data,20)

pred = predict(best_split$xgb_best, best_split$best_down_test[,-dim(best_split$best_down_test)[2]])

caret::confusionMatrix(pred , best_split$best_down_test$Class)

accur_in_test = mean(as.data.frame(caret::confusionMatrix(pred , best_split$best_down_test$Class)$byClass)[,11])

#

datasplit <- reproduce_split(cisdb_fpr001data,best_split$best_seednumber)

pred = predict(best_split$xgb_best, data.matrix(cisdb_fpr001data[,datasplit$colsort]))

accur_in_alldata = mean(as.data.frame(caret::confusionMatrix(pred, cisdb_fpr001data$y)$byClass)[,11])

caret::confusionMatrix(pred, borutadata$y)

sink("20210316_stat_CISDB_fpr001-Boruta_model.txt")

print(caret::confusionMatrix(pred, data$y))

sink()

#

## Load Search results of CIS-DB motifs (by search_pwm.py)

data_cisdb_fpr005 <- read.csv("./motifcount_CISBP_fpr005.csv",header=T)

colnames(data_cisdb_fpr005)[1] <- "y"

data_cisdb_fpr005$y <- as.factor(class)

indx <- 2:length(colnames(data_cisdb_fpr005))

data_cisdb_fpr005[indx] <- lapply(data_cisdb_fpr005[indx], function(x) as.integer(x))

Boruta(y~.,data=data_cisdb_fpr005,

maxRuns = 500,doTrace=2)->Bor.son.cisdb_fpr005

# Select "Confirmed" motifs by Boruta

selected_boruta <- rownames(attStats(Bor.son.cisdb_fpr005))[which(attStats(Bor.son.cisdb_fpr005)$decision == "Confirmed")]

cisdb_fpr005data <- select(.data = data_cisdb1, all_of(c("y", selected_boruta)) )

## Construction and test model

best_split <- estimate_data_split(cisdb_fpr005data,20)

pred = predict(best_split$xgb_best, best_split$best_down_test[,-dim(best_split$best_down_test)[2]])

caret::confusionMatrix(pred , best_split$best_down_test$Class)

accur_in_test = mean(as.data.frame(caret::confusionMatrix(pred , best_split$best_down_test$Class)$byClass)[,11])

# 53%

datasplit <- reproduce_split(cisdb_fpr005data,best_split$best_seednumber)

pred = predict(best_split$xgb_best, data.matrix(cisdb_fpr005data[,datasplit$colsort]))

accur_in_alldata = mean(as.data.frame(caret::confusionMatrix(pred, cisdb_fpr005data$y)$byClass)[,11])

caret::confusionMatrix(pred, borutadata$y)

sink("20210316_stat_CISDB_fpr005-Boruta_model.txt")

print(caret::confusionMatrix(pred, data$y))

sink()

# 55%
