# Supplementary material for: Elucidation of Novel cis-Regulatory Elements and Promoter Structures Involved in Iron Excess Response Mechanisms in Rice Using a Bioinformatics Approach
Source: Front Plant Sci. 2021 Jun 2;12:660303. doi: 10.3389/fpls.2021.660303 (PMC8207140; doi:10.3389/fpls.2021.660303)
Supplement: Supplementary file 1 [file Data_Sheet_1.zip › Supplementary Data 4.docx]

**Supplementary Data 4**

#########################################################

# Code for Modeling of gene expression patterns (Kakei et al. 2021)

#########################################################

## Load fasta file (this inculdes clustered genes of Fe-excess regulon) to search CIS-BP motifs.

from Bio import SeqIO

from Bio.SeqRecord import SeqRecord

file_in ='../200623ModelingClusteredUnnamedNoResponse2.fasta'

seq_records = list(SeqIO.parse(open(file_in, mode='r'), 'fasta'))

## To search motifs using PWM and PSSM score, Background GC content in "-500 to +150" seauence was calculated.

import statistics

def getGC(seq):

return((sum([1.0 for nucl in seq if nucl in ['G', 'C']]) / len(seq)) * 100)

file_in ='../promoter-ratio-500+150_Rice44k-ExcessFe.fasta'

seq_all = list(SeqIO.parse(open(file_in, mode='r'), 'fasta'))

gc_contents = [getGC(seq) for seq in seq_all]

statistics.mean(gc_contents)

# 48.19

## Load motifs from CIS-BP (http://cisbp.ccbr.utoronto.ca/)

## All the CIS-BP rice motifs were downloaded as Oryza_sativa_2021_03_04_1 33_am.zip amd decompressed.

## (*) In Scan single sequences for TF binding tool, sample search parameter was set as PWMs - LogOdds = 8

from Bio import motifs

import os

import pandas as pd

motiffilelist = os.listdir('./Oryza_sativa_2021_03_04_1 33_am/pwms_all_motifs')

## Search PWM notifs using precision and balanced threshold

# distribution = pssm.distribution(background=background, precision=10**4)

# threshold = distribution.threshold_balanced(1000)

def search_motifs(file):

filename = "{}/{}".format('./Oryza_sativa_2021_03_04_1 33_am/pwms_all_motifs',file)

found=0

if os.path.getsize(filename) < 13:

return found

with open(filename) as handle:

motif = motifs.read(handle, 'pfm-four-columns')

#print(motif.counts)

pwm = motif.counts.normalize(pseudocounts={"A":0.52, "C": 0.48, "G": 0.48, "T": 0.52})

#print(pwm)

pssm = pwm.log_odds(background)

#print(pssm)

#print("%4.2f" % pssm.max)

#print("%4.2f" % pssm.min)

#mean = pssm.mean(background)

#std = pssm.std(background)

#print("mean = %0.2f, standard deviation = %0.2f" % (mean, std))

distribution = pssm.distribution(background=background, precision=10**4)

threshold = distribution.threshold_balanced(1000)

for position, score in pssm.search(test_seq, threshold=threshold):

#print("Motif_ID %s Position %d: score = %5.3f" % (file,position, score))

found=1

return found

from joblib import Parallel, delayed

background = {'A':0.26,'C':0.24,'G':0.24,'T':0.26}

dfObj = pd.DataFrame(columns=motiffilelist)

n=0

for test_seq in seq_records:

# These comment-outed commands are for non-parallel calculation

# list = []

# for file in motiffilelist:

# filename = "{}/{}".format('./Oryza_sativa_2021_03_04_1 33_am/pwms_all_motifs',file)

# found=search_motifs(file)

# list.append(found)

#motiffilelist_small = motiffilelist[:5]

#list = Parallel(n_jobs=-1)([delayed(search_motifs)(file) for file in motiffilelist])

dfObj.loc[n] = Parallel(n_jobs=-1)([delayed(search_motifs)(file) for file in motiffilelist])

n=n+1

dfObj.to_csv("motifcount_CISBP.csv")

## Another method to determine threshold as 80% value to max_score

def search_motifs2(file):

filename = "{}/{}".format('./Oryza_sativa_2021_03_04_1 33_am/pwms_all_motifs',file)

found=0

if os.path.getsize(filename) < 13:

#list.append(found)

#continue

return found

with open(filename) as handle:

motif = motifs.read(handle, 'pfm-four-columns')

#print(motif.counts)

pwm = motif.counts.normalize(pseudocounts={"A":0.52, "C": 0.48, "G": 0.48, "T": 0.52})

#print(pwm)

pssm = pwm.log_odds(background)

max_score = pssm.max

min_score = pssm.min

abs_score_threshold = (max_score - min_score) * 0.8 + min_score

for test_seq in seq_records:

motif.pseudocounts = motifs.jaspar.calculate_pseudocounts(motif)

for position, score in pssm.search(test_seq,threshold=abs_score_threshold):

rel_score = (score - min_score) / (max_score - min_score)

print("Position %d: score = %5.3f, rel. score = %5.3f" % (position, score, rel_score))

found=1

#list.append(found)

return found

dfObj = pd.DataFrame(columns=motiffilelist)

n=0

for test_seq in seq_records:

# These comment-outed commands are for non-parallel calculation

# list = []

# for file in motiffilelist:

# filename = "{}/{}".format('./Oryza_sativa_2021_03_04_1 33_am/pwms_all_motifs',file)

# found=search_motifs(file)

# list.append(found)

#motiffilelist_small = motiffilelist[:5]

#list = Parallel(n_jobs=-1)([delayed(search_motifs)(file) for file in motiffilelist])

dfObj.loc[n] = Parallel(n_jobs=-1)([delayed(search_motifs)(file) for file in motiffilelist])

n=n+1

dfObj.to_csv("motifcount_CISBP2.csv")
