# Supplementary material for: Elucidation of Novel cis-Regulatory Elements and Promoter Structures Involved in Iron Excess Response Mechanisms in Rice Using a Bioinformatics Approach
Source: Front Plant Sci. 2021 Jun 2;12:660303. doi: 10.3389/fpls.2021.660303 (PMC8207140; doi:10.3389/fpls.2021.660303)
Supplement: Supplementary file 1 [file Data_Sheet_1.zip › Supplementary Figures.pdf]

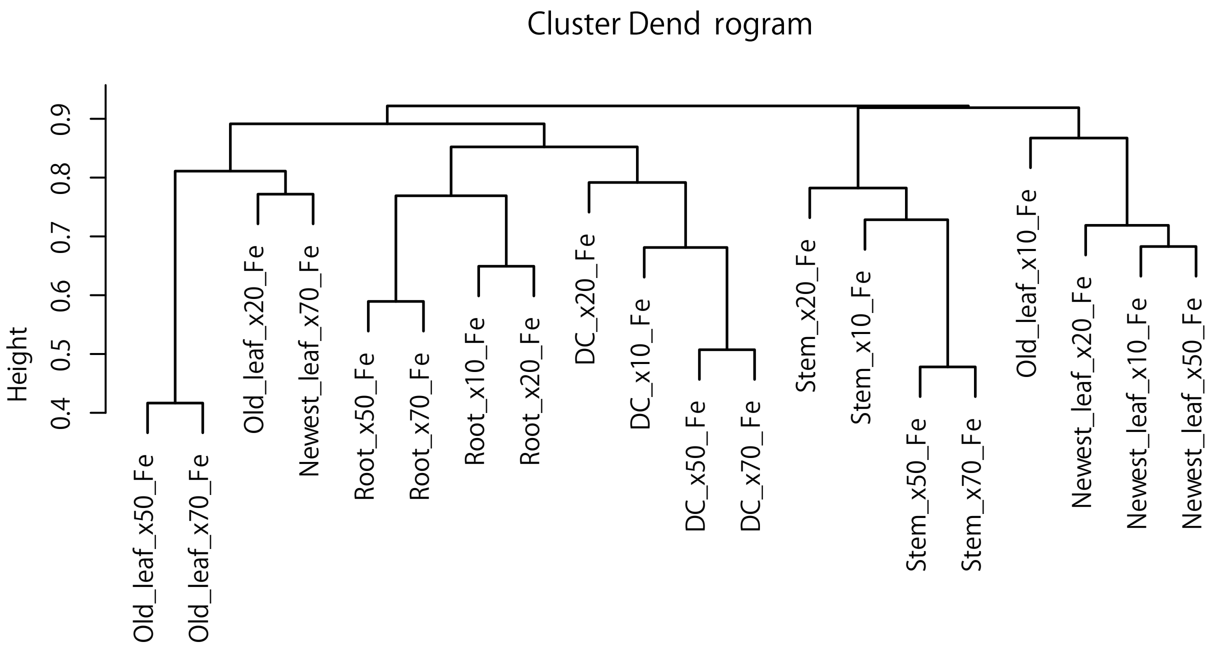

SUPPLEMENTARY FIGURE 1 | Cluster dendrogram of Fe excess microarray data.
Gene expression patterns obtained using Fe excess microarray data from various rice tissues under various levels of Fe excess of ×10, ×20, ×50 and ×70 Fe compared to the control (×1 Fe) were analyzed and clustered into a tree model. Clusters positioned near each other show more similar gene expression patterns. DC: discrimination center, or junction node between the root and shoot. 10, 20, 50, 70, indicate 10-fold, 20-fold, 50-fold, and 70-fold excess Fe levels.

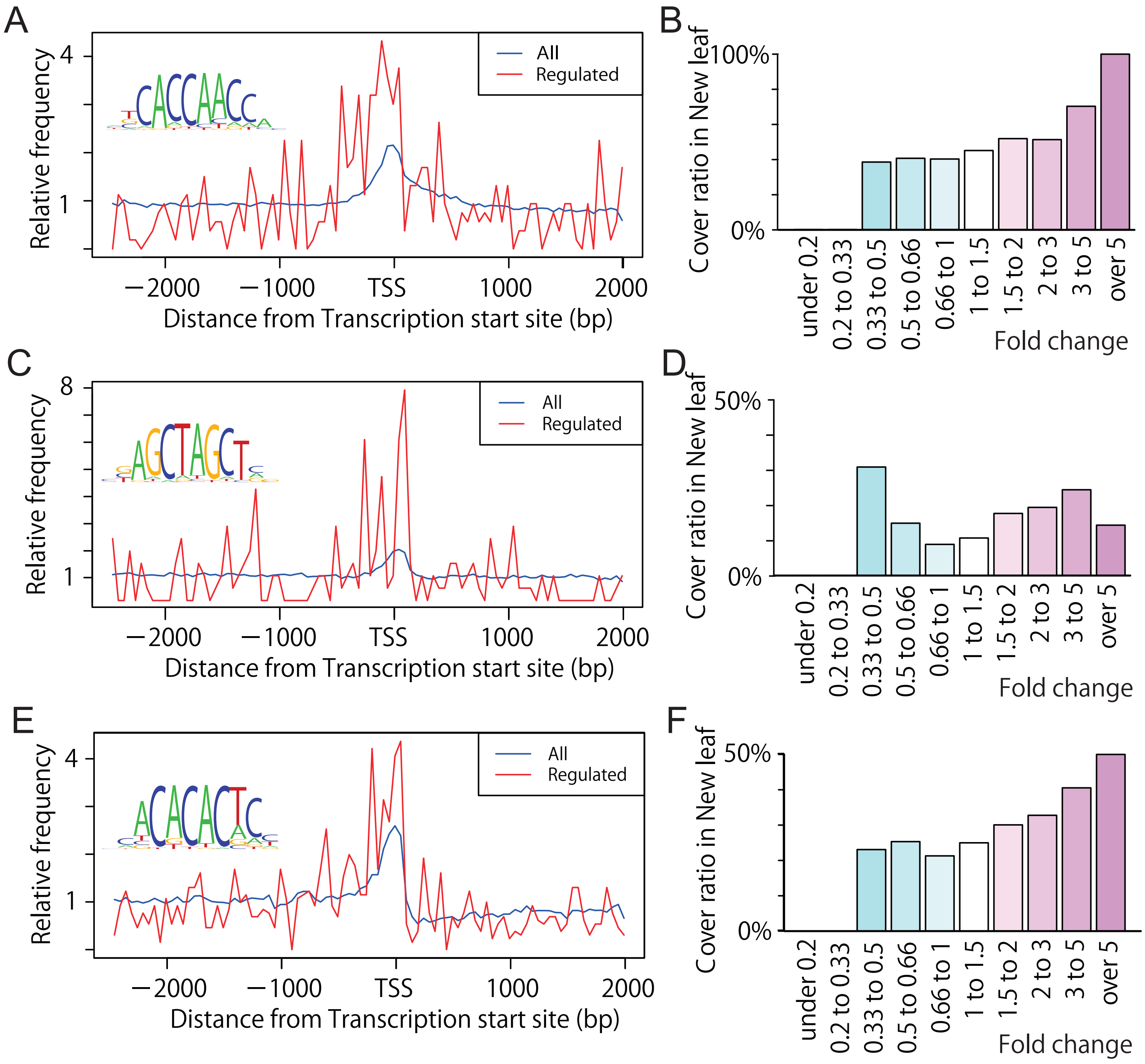

SUPPLEMENTARY FIGURE 2 | Distribution of MAMA motifs in upstream sequences of Fe excess-responsive genes in the newest leaf.
(A) Distribution of the CACCAACC (novel) motif in all genes and in Fe excess-regulated genes of the newest leaf. Blue line shows all genes and red line shows the Fe excess-regulated genes. **(B)** Coverage ratio (percentage of genes including motif among the which up- or down-regulated genes by Fe excess as fold changes described under graph) of the CACCAACC motif in the 500 bp region upstream of the TSS. **(C)** Distribution of the FAM1 (AGCTAAGCT) motif. **(D)** Coverage ratio of the AGCTAAGCT motif. **(E)** Distribution of the ACACACTC (novel) motif. **(F)** Coverage ratio of the ACACACTC motif. Graphs (A, C, E) show relative frequency compared to the average frequency in each 50 bp window within -3000 bp to +2000 bp of the transcription start site (TSS). Graphs (B, D, F) show coverage ratio of the motifs in the 500 bp region upstream of the TSS in Fe excess-treated newest leaves. Numbers under the graph in B, D, and F indicate gene expression ratios in the newest leaf of Fe excess-treated rice relative to non-treated rice.

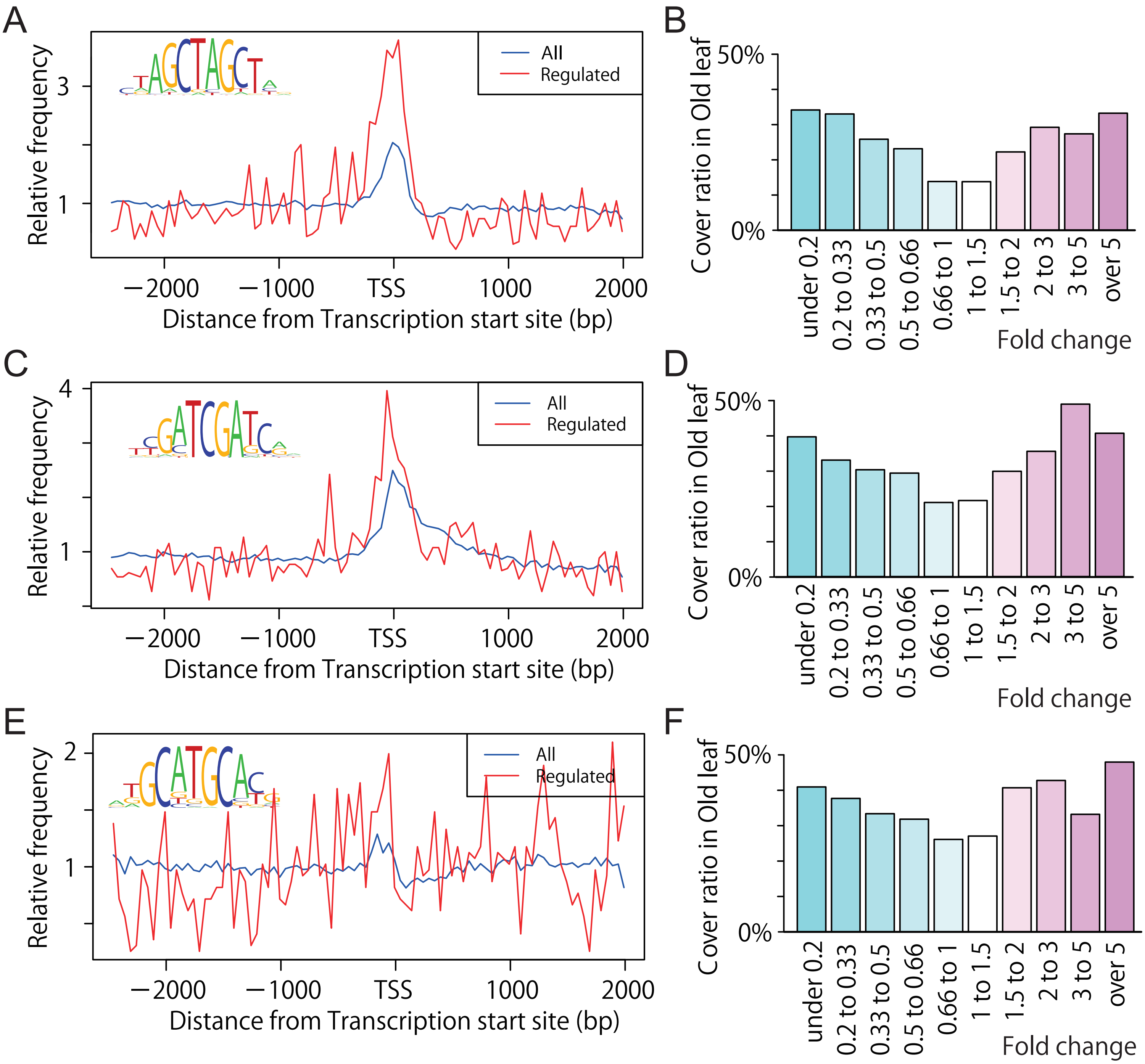

SUPPLEMENTARY FIGURE 3 | Distribution of MAMA motifs upstream of Fe excess-responsive genes in old leaf tissue. **(A)** Distribution of the FAM1 (AGCTAGCT) motif in all genes and in Fe excess-regulated genes in old leaf. Blue line shows all genes and red line shows the Fe excess-regulated genes. **(B)** Coverage ratio (percentage of genes including motif among the up- or down-regulated genes by Fe excess as fold changes described under graph) of the AGCTAGCT motif in the region 500 bp upstream of the TSS. **(C)** Distribution of the GATCGATC (novel) motif. **(D)** Coverage ratio of the GATC-GATC motif. **(E)** Distribution of the GCATGCAC (novel) motif. **(F)** Coverage ratio of the GCATGCAC motif. Graphs (A, C, E) show relative frequency compared to the average frequency in each 50 bp window within -3000 bp to +2000 bp of the transcription start site (TSS). Graphs (B, D, F) show coverage ratio of the motifs in the 500 bp region upstream of the TSS in Fe excess-treated old leaves. Numbers under the graph in B, D, and F represent gene expression ratios in old leaves of Fe-excess-treated rice compared to non-treated rice.

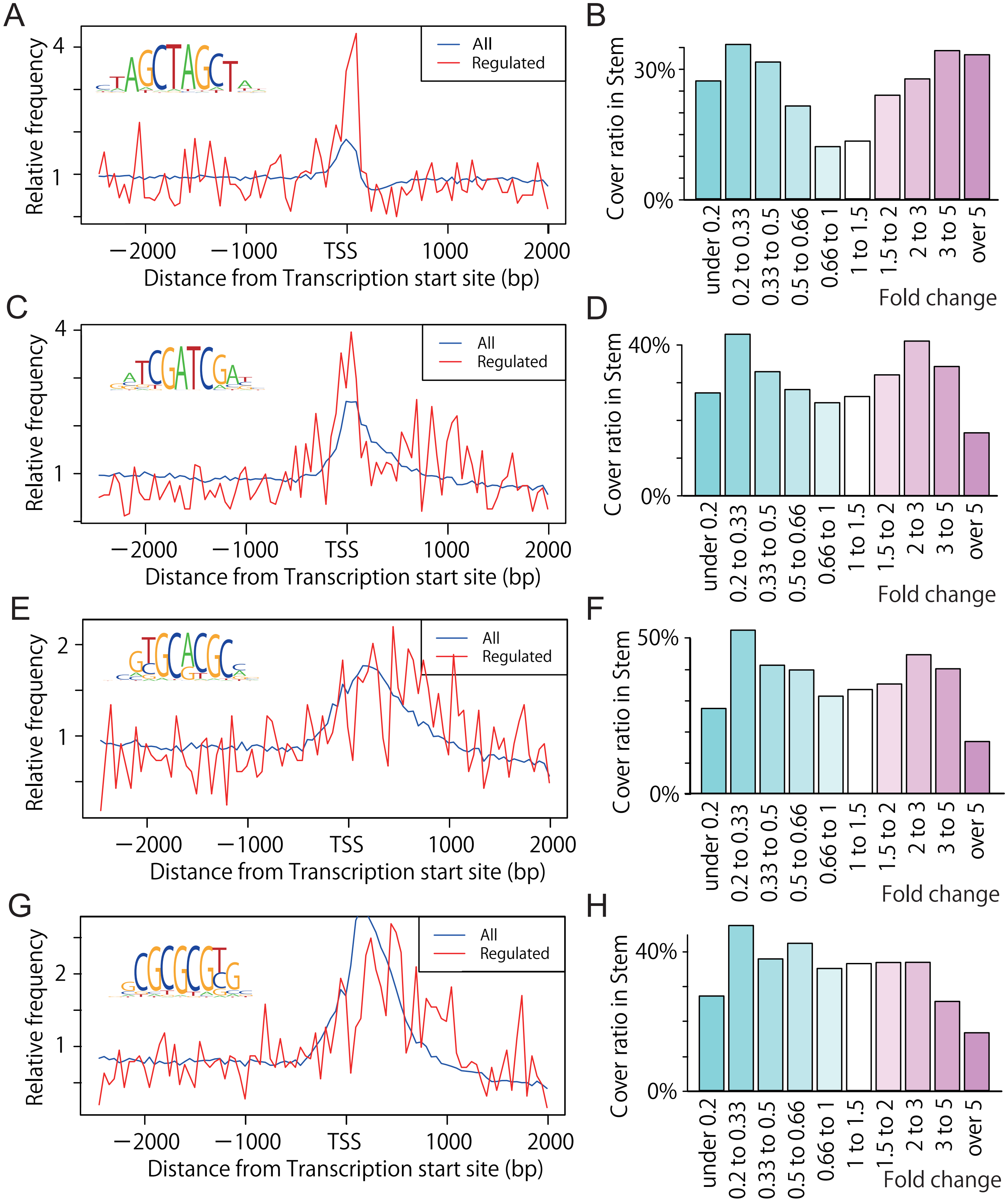

SUPPLEMENTARY FIGURE 4 | Distribution of MAMA motifs upstream of Fe excess-responsive genes in the stem. **(A)** Distribution of the FAM1 (AGCTAGCT) motif in all genes and in Fe excess-regulated genes in the stem. Blue line shows all genes and red line shows the Fe excess-regulated genes. **(B)** Coverage ratio (percentage of genes including motif among the up- or down-regulated genes by Fe excess as fold changes described under graph) of the AGCTAGCT motif in the area 500 bp upstream of the TSS. **(C)** Distribution of the TCGATCGA (novel) motif. **(D)** Coverage ratio of the TCGATCGA motif. **(E)** Distribution of the TGCACGC (novel) motif. **(F)** Coverage ratio of the TGCACGC motif. **(G)** Distribution of the CGCGCGTG (novel) motif. **(H)** Coverage ratio of the CGCGCGTG motif. Graphs (A, C, E, G) show relative frequency compared to the average frequency in each 50 bp window within -3000 bp to +2000 bp of the transcription start site (TSS). Graphs (B, D, F, H) show coverage ratio of the motifs in the 500 bp region upstream of the TSS in Fe excess-treated stems. Numbers under the graph in B, D, F, and H are gene expression ratios in the stem of Fe excess-treated rice compared to non-treated rice.

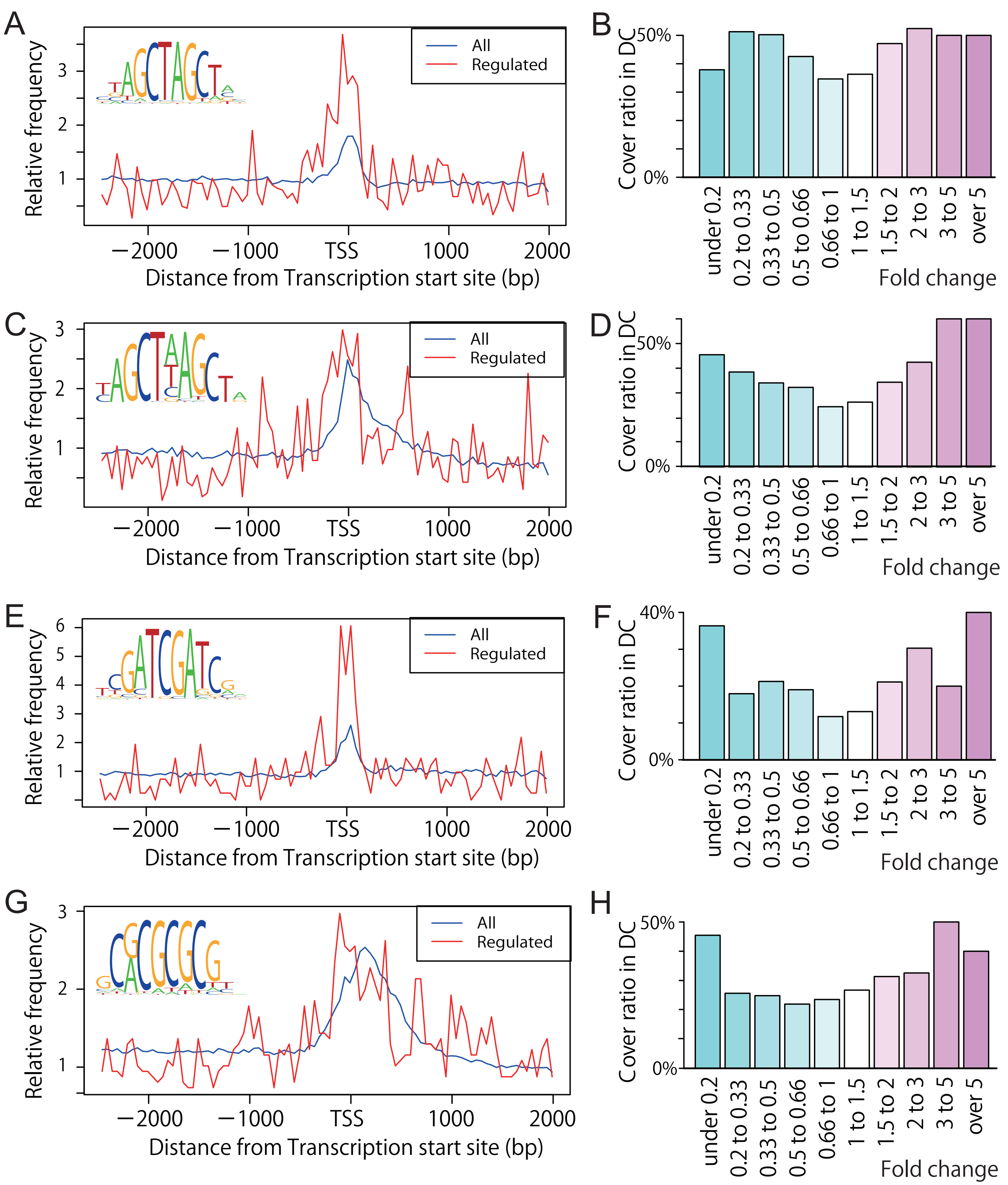

SUPPLEMENTARY FIGURE 5 | Distribution of MAMA motifs upstream of Fe excess-responsive genes in the DC. **(A)** Distribution of the FAM1 (AGCTAGCT) motif in all genes and in Fe excess-regulated genes in the DC. Blue line shows all genes and red line shows the Fe excess-regulated genes. **(B)** Coverage ratio (percentage of genes including motif among the up- or down-regulated genes by Fe excess as fold changes described under graph) of the AGCTAGCT motif in the region 500 bp upstream of the TSS. **(C)** Distribution of the AGCTAAGCT (novel) motif. **(D)** Coverage ratio of the AGCTAAGCT motif. **(E)** Distribution of the GATCGATC (novel) motif. **(F)** Coverage ratio of the GATCGATC motif. **(G)** Distribution of the CGCGCGCG motif (novel). **(H)** Coverage ratio of the CGCGCGCG motif. Graphs (A, C, E, G) show relative frequency compared to the average frequency in each 50 bp window within -3000 bp to +2000 bp of the transcription start site (TSS). Graphs (B, D, F, H) show coverage ratio of the motifs in the 500 bp region upstream of the TSS in Fe excess-treated DCs. Numbers under the graph in B, D, F, and H are gene expression ratios in the DC of Fe excess-treated rice compared to non-treated rice. DC: Descrimination center or the junction nodes between root and shoot.

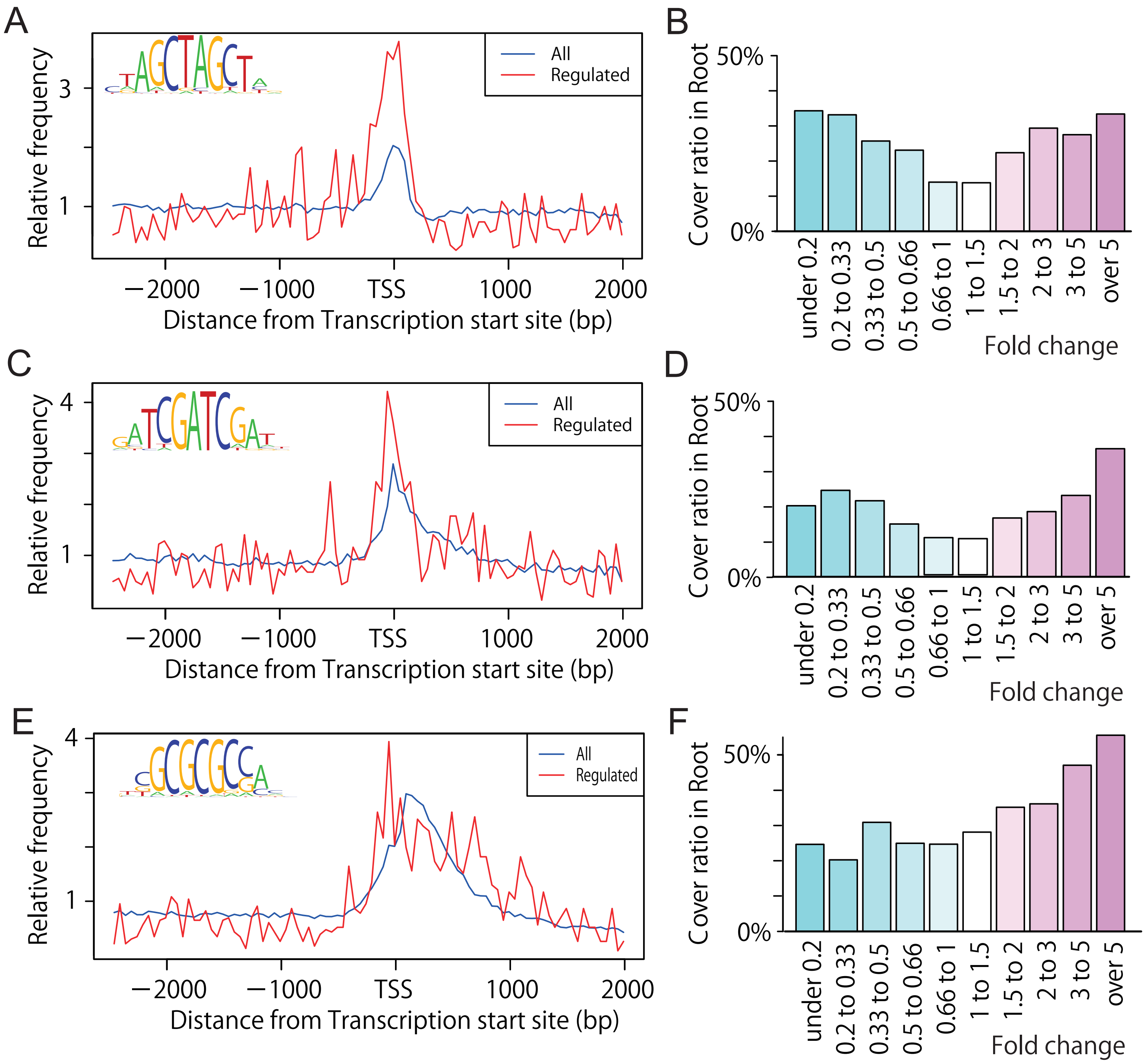

SUPPLEMENTARY FIGURE 6 | Distribution of MAMA motifs upstream of Fe excess-responsive genes in the roots. **(A)** Distribution of the FAM1 (AGCTAGCT) motif in all genes and in Fe excess-regulated genes in the root. Blue line shows all genes and red line shows the Fe excess-regulated genes. **(B)** Coverage ratio (percentage of genes including motif among the up- or down-regulated genes by Fe excess as fold changes described under graph) of the FAM1 (AGCTAGCT) motif in the area 500 bp upstream of the TSS. **(C)** Distribution of the TCGATCGA motif (novel). **(D)** Coverage ratio of the TCGATCGA motif. **(E)** Distribution of the GCGCGCC motif (E2F binding-like). **(F)** Coverage ratio of the GCGCGCC motif. Graphs (A, C, E) show relative frequency compared to the average frequency in each 50 bp window within -3000 bp to +2000 bp of the transcription start site (TSS). Graphs (B, D, F) show coverage ratio of the motifs in the 500 bp region upstream of the TSS in Fe excess-treated roots. Numbers under the graph in B, D, and F are gene expression ratios in the Fe excess-treated rice root compared to non-treated rice.
